# Piecewise parametric structure in the pooling problem: from sparse strongly-polynomial solutions to NP-hardness

**DOI:** 10.1007/s10898-017-0577-y

**Published:** 2017-10-25

**Authors:** Radu Baltean-Lugojan, Ruth Misener

**Affiliations:** Department of Computing, Imperial College London, 180 Queens Gate, London, SW7 2AZ UK

**Keywords:** Standard pooling problem, Global optimization, Piecewise structure, Sparsity, Discretization, *P* / *NP* boundary, Strongly-polynomial algorithms

## Abstract

The standard pooling problem is a NP-hard subclass of non-convex quadratically-constrained optimization problems that commonly arises in process systems engineering applications. We take a parametric approach to uncovering topological structure and sparsity, focusing on the single quality standard pooling problem in its *p*-formulation. The structure uncovered in this approach validates Professor Christodoulos A. Floudas’ intuition that pooling problems are rooted in piecewise-defined functions. We introduce dominant active topologies under relaxed flow availability to explicitly identify pooling problem sparsity and show that the sparse patterns of active topological structure are associated with a piecewise objective function. Finally, the paper explains the conditions under which sparsity vanishes and where the combinatorial complexity emerges to cross over the *P* / *NP* boundary. We formally present the results obtained and their derivations for various specialized single quality pooling problem subclasses.

## Introduction

The standard pooling problem represents a NP-hard subclass [3] of non-convex quadratically-constrained optimization problems with bilinear terms and may have a multiplicity of local minima [33]. Pooling problems model the computational difficulties associated with intermediate blending of heterogeneous feedstocks and therefore have direct application in process system engineering [34, 56]. Specific application domains include: petroleum refining [7, 20, 49], mining [14], wastewater treatment [35, 42], crude oil scheduling [39], natural gas production [50], etc. We recently showed that standard pooling arises as a sub-problem pattern in general mixed-integer nonlinear optimization (MINLP) [17].

Motivated by applications, Floudas and Visweswaran [22, 23, 57, 58] were the first to rigorously solve the pooling problem to global optimality. The Floudas and Visweswaran approach uses duality theory and Lagrangian relaxations. Subsequent global optimization contributions to solving the pooling problem include: making further Lagrangian relaxation contributions [1], developing alternative problem formulations [5, 11], augmenting models with reformulation-linearization cuts [42, 48, 51, 52] to create a provably dominant formulation [53], developing problem-specific polyhedral cuts based on small pooling networks [18, 19], and identifying the *P* / *NP* boundary with respect to the topological structure [3, 15, 31, 32]. More general techniques for non-convex quadratically-constrained optimization problems with bilinear terms are also appropriate for the pooling problem. The more general methods include: using convex envelopes to formulate a linear relaxation [2, 24, 41], developing a general branch-and-cut method [6], applying a sum-of-squares hierarchy [40], and using state-of-the-art global optimization MINLP solver software [10, 12, 13, 37, 45, 46, 53–55]. Further details are available in reviews discussing the pooling problem [5, 16, 30, 43].

But, despite significant attention to the pooling problem, deterministic global optimization algorithms can have significant optimality gaps and impractical or unknown convergence times on large-scale, industrially-relevant instances. These impractical convergence properties are interesting because Beale et al. [9] report that a simple, piecewise-linear program serves as a practical heuristic for small, pooling-like instances. Meyer and Floudas [42] had a similar intuition that very large pooling problems may be approached via piecewise-linear relaxation schemes. This intuition, which also appeared in Karuppiah and Grossmann [35], suggests that the pooling problem, a continuous nonlinear optimization problem (NLP), may be effectively approximated as a mixed-integer linear optimization problem (MILP). Further evidence for this intuition appears in several effective algorithms optimizing industrially-relevant pooling instances via piecewise-linear approaches, e.g. [26, 29, 36, 44, 47, 59]. Subsequent work used the standard pooling problem topology to develop a state-of-the-art MILP discretization heuristic with a performance bound [21, 27, 28].

**Fig. 1:**
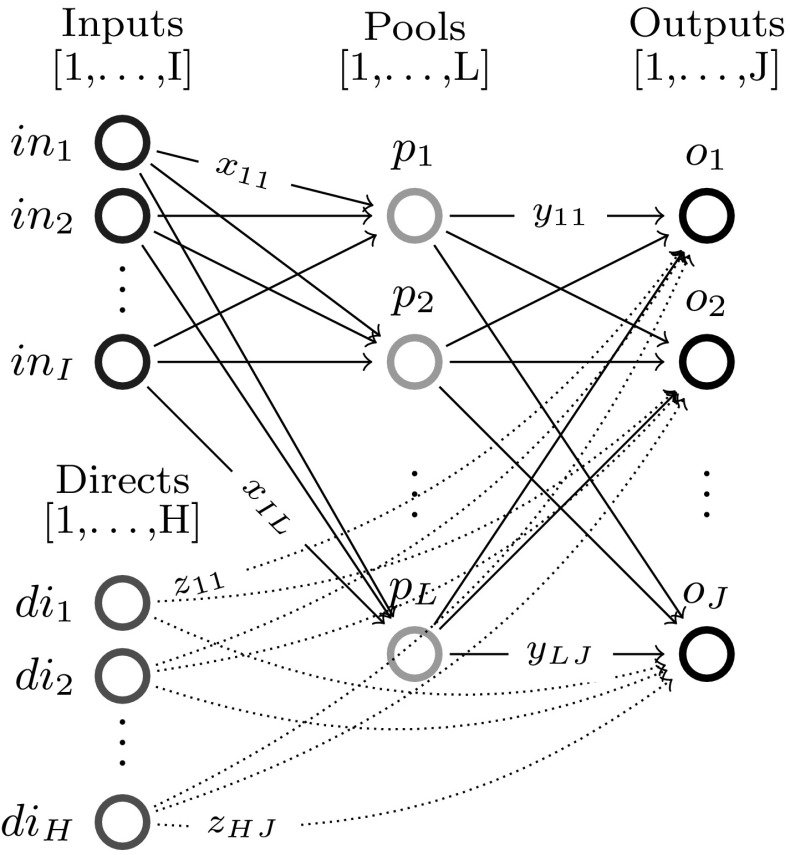
Standard pooling network. Note the feeds layer is separated into two groups of nodes: input nodes [1,...,I] that send flows to pools only and direct nodes [1,...,H] that send flows to outputs only. In general, one feed can send flows both to pools and outputs. In this case, any of the feed’s flows is assigned, based on the layer they are sent towards, to either an input or direct node corresponding to the feed. This explicit input/direct separation helps build a clear understanding of the problem sparsity/structure as related to flows to pools versus flows to outputs. The separation is generally adopted in the paper’s figures, discussions or proofs, as needed

This paper validates and substantiates Professor Floudas’ intuition by formalizing and characterizing the piecewise structures arising in standard pooling subclasses. We build a bottom-up, intuitive understanding of the *P* / *NP* boundary of the single quality standard pooling problem by taking a parametric view and relaxing the flow availability box constraints. The relaxations employed effectively remove the flow availability bounds on feeds and pools and fix the product demand at each output. In the semantics of Boland et al. [15], e.g. Fig. 1 of their manuscript, our approach unifies and generalizes the $|K| = 1$ complexity results. Our parametric approach yields polynomial-time subclasses with a piecewise-linear or piecewise convex/concave monotone structure. We formalize these piecewise structures in single quality standard pooling subclasses that offer exact global solutions in polynomial time. The proofs lead to the unexpected outcome that the famous Haverly [33] pooling instances, i.e. the first-recorded pooling instances, belong to a strongly-polynomial subclass! The strongly-polynomial result for the Haverly [33] instances is remarkable because these case studies have been used as test cases for exponential algorithms for more than 35 years.

This manuscript also justifies the Beale et al. [9] observation that the linear approximation is most effective when only a few variables are active at once. Using patterns of dominating topologies, we explicitly identify pooling problem sparsity, i.e. a limited number of active flow variables. We show that these sparse patterns of active topological structure are associated with a piecewise objective function and we take advantage of these structures. Lastly, we explain the conditions under which such sparsity vanishes by reintroducing constraints on flow availability and, together with them, the combinatorial complexity needed to cross over the *P* / *NP*-time boundary.

The paper proceeds as follows: Section 2 introduces the single quality formulation of the standard pooling problem and the assumptions (flow constraint relaxations) used throughout this paper; Sect. 3 analyzes the one pool, one output subclass and uncovers both a piecewise-monotone structure and a strongly-polynomial time algorithm for solving it; Sect. 4 extends the results in Sect. 3 to the subclass with multiple outputs via additive decomposition over outputs; Sect. 5 extends Sect. 3 results to the subclass with multiple pools using problem sparsity; Sect. 6 discusses the implications and possible extensions of the results. The source code implementation of the results discussed in this paper is available on Github [8].

## Standard pooling *p*-formulation and assumptions used

This manuscript unpacks single quality standard pooling problem solutions by parameterizing with respect to pool concentrations. To effectively do so, the paper employs a concentration-based formulation, i.e. the *p*-formulation shown in Problem P–2.1 [5]. Table 1 introduces the notation used for indices, sets, variables, parameters, as well as for the problem subclass types analyzed in the following sections. Fig. 1 shows the topological structure of a standard pooling network, represented by a feed-forward flow network of 3 node layers.

**Table 1:** Standard pooling problem notation [43]

Type	Notation	Description
Indices	$i \in \{i \ |\ (i,\cdot )\in T_X \cup T_Z\}$	Input streams (raw materials or feed stocks)
	$l \in \overline{1,L}$	Pools (blending facilities)
	$j \in \overline{1,J}$	Output streams (end products)
	$k \in \overline{1,K}$	Attributes (qualities monitored)
Sets	$T_X$	(*i*, *l*) pairs for which input to pool connection exists, $|T_X|=I$
	$T_Z$	(*i*, *j*) pairs for which input to output connection exists, $|T_Z|=H$
	$T_Y$	(*l*, *j*) pairs for which pool to output connection exists
Problem type	$I{+}H{-}L{-}J{-}K$	$I{+}H$ feeds (inputs + directs), *L* pools, *J* outputs and *K* qualities
	$I{+}H{-}L{-}J$	$I{+}H$ feeds (inputs + directs), *L* pools, *J* outputs and one quality
	$I{-}L{-}J$	(No directs) *I* inputs, *L* pools, *J* outputs and one quality
	$H{-}L{-}J$	(No inputs) *H* directs, *L* pools, *J* outputs and one quality
Variables	$x_{i,l}$	Flow from input *i* to pool *l*
	$y_{l,j}$	Flow from intermediate pool node *l* to output *j*
	$z_{i,j}$	Bypass flow directly from input feed stock *i* to product *j*
	$p_{l,k}$	Level/concentration of quality attribute *k* in pool *l*
Parameters	*f*	The objective function of the problem
	$\gamma _i$	Unit cost of raw material feed stock *i*
	$d_j$	Unit revenue for product *j*
	$A_i^L-A_i^U$	Availability bounds (required usage to max. availability) of input *i*
	$S_l$	Volumetric size capacity of pool *l*
	$D_j^L-D_j^U$	Demand bounds (required to limit demand) for product *j*
	$C_{i,k}$	Level of quality *k* in raw material feed stock *i*
	$P_{j,k}^L-P_{j,k}^U$	Acceptable composition range of quality *k* in product *j*

The standard pooling problem is a flexible formulation used for many real-world applications with different measure units for variables/parameters, e.g. in [35]: flows(ton/hr), quality level/concentration(ppm), objective/unit cost/unit revenue($), capacity(tons)

Different flows pass between the three layers, having different concentrations of various qualities, e.g. crude oil chemical compositions. Input feeds $in_1 - in_I$ send flows denoted by *x* variables to be linearly blended in *L* pools ($p_1 - p_L$), that further distribute *y* flows to *J* outputs ($o_1 - o_J$) to create blended products. Additionally, *H* direct feeds $di_1 - di_H$ send *z* flows directly to the outputs layer. The standard pooling Problem P–2.1 consists of maximizing a profit function with profits and costs associated to each network flow, subject to flow constraints, e.g. feed availability, pool capacity, output demands, flow balance at pools, and quality concentration constraints, e.g. quality balance at pools, product quality bounds at outputs. This manuscript addresses a somewhat more complex pooling problem than previous work analyzing the *P* / *NP* boundary [3, 15, 31, 32] by considering the direct feeds $di_1 - di_H$. 
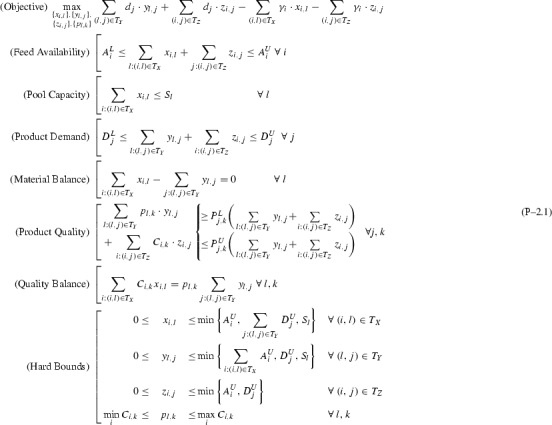


### Remark 2.1

In Problem P–2.1, the upper hard bounds on variable sets $\{x\},\ \{y\},\ \{z\}$ are redundant and can be dropped. These upper hard bounds are implicitly met by simultaneously enforcing the constraints on feed availability, pool capacity, product demand and material balance. Similarly, the hard bounds on $p_{l,k}\ \forall l,k$ can be dropped, as they are implicitly met by replacing all *y* variables in the quality balance with *x* variables from the material balance.

### Assumption 2.2

Problem P–2.1 is restricted to a single quality and assumed feasible, with dropped constraints on feed availability and pool capacity and fixed product demands $D_j>0,\ \forall j$.

***Note:*** Remarks 3.3.10, 4.6 and 5.6 explain how Assumption 2.2 provides tight bounds for sparsity and polynomial-time solvability. Remark 3.3.10 shows that the sparse, piecewise monotone structure of subclass $\mathrm{I}{+}\mathrm{H}{-}1{-}1$ is tightly conditioned on Assumption 2.2. Remark 4.6 shows that the polynomial-time solvability of subclass $\mathrm{I}{+}\mathrm{H}{-}1{-}\mathrm{J}$ is also tightly conditioned on Assumption 2.2. Remark 5.6 justifies these observations for the $\mathrm{I}{+}\mathrm{H}{-}\mathrm{L}{-}1$ subclass.

Removing the feasibility assumption is not discussed, at it serves only to remove the check for an infeasible/unprofitable problem with all feeds inactive and $f^*=0$.

Based on Remark 2.1 and Assumption 2.2, after dropping indices *k* for simplicity and denoting the objective function with *f*, Problem P–2.1 becomes: 
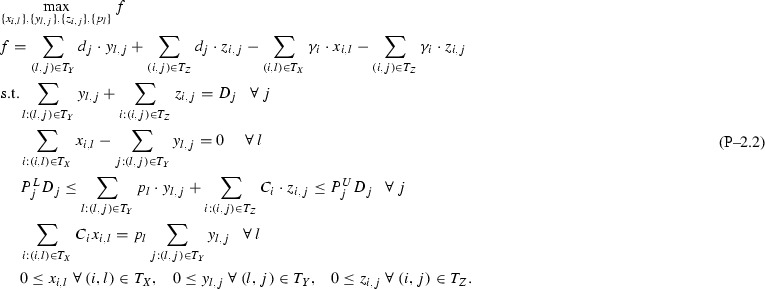
 The following sections analyze bottom-up several subclasses of Problem P–2.2 based on topological restrictions, proving in each case strongly-polynomial time complexity coupled with finding sparse piecewise structures. The analysis contours the *P* / *NP*-hard boundary for Problem P–2.2 subclasses.

## Subclass $\mathrm{I}{+}\mathrm{H}{-}1{-}1$: one pool, one output

This section analyzes the topological restriction of Problem P–2.2 with $I{+}H$ feeds (*I* inputs, *H* directs), one pool, and one output. For simplicity of notation, single indices *l* and *j* are dropped from variables and parameters via the notation transformations $T_Z \leftarrow \{i:(i,j)\in T_Z\}$, $T_X \leftarrow \{i:(i,l)\in T_X\}$ leading to the restricted Problem P–3.3. 
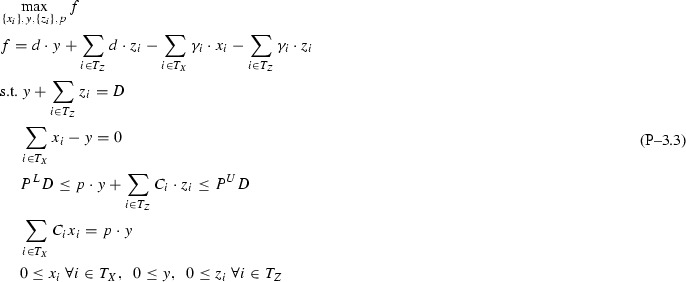
 
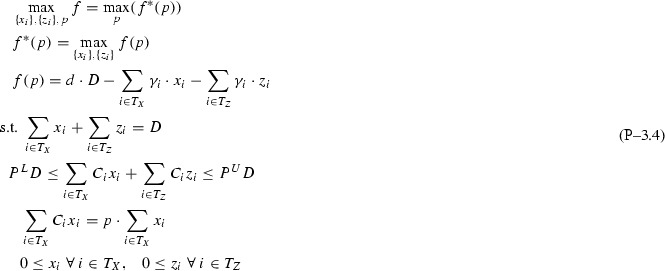


Variables *p*, *y* can be eliminated from Problem P–3.3 by directly substituting all $y,\ p\cdot y$ terms from their constraints. Thus, *f* can be rewritten:

$$\begin{aligned} f&= d\cdot y+\sum _{i\in T_Z}d\cdot z_{i} -\sum _{i\in T_X}\gamma _i\cdot x_{i}-\sum _{i\in T_Z}\gamma _i\cdot z_{i} = \sum _{i\in T_X}(d-\gamma _i)\cdot x_{i}+\sum _{i\in T_Z}(d-\gamma _i)\cdot z_{i}\\&=d\cdot D -\sum \limits _{i\in T_X}\gamma _i\cdot x_{i}-\sum \limits _{i\in T_Z}\gamma _i\cdot z_{i}. \end{aligned}$$

In Problem P–3.4, we eliminate *y* but retain *p* as a parameter controlling flows $x_i,\ \forall i\in T_X$ relative to each other. In addition, eliminating *p* from Problem P–3.3 produces a linear program (LP) in the $x,\ z$ variables. Since at optimality the LP has at most three tight constraint bounds (product quality can only have one tight bound), the cardinality of the optimal basis is at most three, but the basic variables among $x,\ z$ can not be identified directly in this manner. Consequently, retaining parameter *p* allows us to analytically understand the optimal solutions for Problem P–3.4 and identify basic variables among $x,\ z$ across *p*-intervals, together with any problem structure. In particular, the objective function *p*-parametric form may be used to break the *p*-interval $[\min _{i} C_{i},\ \max _{i} C_{i}]$ into sub-intervals where special properties of *f* arise. Section 4 uses this parametric approach for solving a non-convex/non-linear problem subclass in strongly-polynomial time.

Active sets, dominance relations and breakpoints are essential building blocks to find the structure of $f^*(p)$ in Problem P–3.4 and are all introduced in Definitions 3.1–3.4.

### Definition 3.1

*(Active sets, objective function and cost function)* Set *A* of nodes from the feed layer is:

- An **input active set** if $A\subseteq T_X,\qquad \ x_i=0\ \forall i \in T_X \setminus A,\ z_i=0\ \forall i \in T_Z$.
- A **direct active set** if $\ A\subseteq T_Z,\qquad \ x_i=0\ \forall i \in T_X,\qquad \,\, z_i=0\ \forall i \in T_Z \setminus A$.
- A **mixed active set** if $A\subseteq T_X\cup T_Z,\ x_i=0\ \forall i \in T_X\setminus A,\ z_i=0\ \forall i \in T_Z \setminus A,\ A\setminus T_X\notin \{A,\emptyset \}$.

For an active set *A* in Problem P–3.4, the objective function *f* is given by,

1 $$\begin{aligned} f= {\left\{ \begin{array}{ll}\begin{array}{llll} f_A(p)&{}=d\cdot D- &{} \sum \limits _{i\in A\cap T_X} \gamma _i x_i - \sum \limits _{i\in A\cap T_Z} \gamma _i z_i,\ &{}\text {if } A \text { is an input or mixed active set},\\ f_A&{}=d\cdot D - &{} \sum \limits _{i\in A} \gamma _i z_i,\ &{}\text {if } A \text { is a direct active set,} \end{array}\end{array}\right. } \end{aligned}$$

where *f* is not *p*-parametric in the second case of no input flow to the pool since pool concentration *p* is undefined. Let $h=d \cdot D-f$ denote the cost function associated with objective function *f*.

### Definition 3.2

*(Feasibility with respect to product quality constraints)* A Problem P–3.4 active set is **feasible** if the product quality bounds $[P^L,P^U]$ are met, i.e. the second constraint holds. An infeasible active set is not a valid Problem P–3.4 solution and is therefore strictly dominated by any feasible active set (see Definition 3.3).

### Definition 3.3

*(Dominance and breakpoints between active sets)* Let $A_1,A_2$ be feasible input/mixed active sets. Let $f^*_A(p)$ be the optimal objective function value to Problem P–3.4 and $h^*_A(p)$ its corresponding optimal cost function value, assuming active set *A* and fixed *p*.

- Set $A_1$ **dominates** $A_2$ **at** *p* (in the sense of maximized objective function profitability) when,
  2 $$\begin{aligned} {\varvec{A}}_1 {\varvec{\succeq }}_p {\varvec{A}}_2 \quad \Leftrightarrow \quad f^*_{A_1}(p) \ge f^*_{A_2}(p) \quad \Leftrightarrow \quad h^*_{A_1}(p) \le h^*_{A_2}(p). \end{aligned}$$
- Pool concentration *p* is a **breakpoint between** $A_1$ **and** $A_2$ if:
  3 $$\begin{aligned} {\varvec{A}}_1 {\varvec{\asymp }}_p {\varvec{A}}_2 \quad \Leftrightarrow \quad f^*_{A_1}(p) = f^*_{A_2}(p) \quad \Leftrightarrow \quad h^*_{A_1}(p) = h^*_{A_2}(p). \end{aligned}$$
- The dominance relation also extends to direct active sets, but in this case *f* is not parametric on *p*. Consequently, when comparing two direct active sets, dominance is established similarly via Eq. (2) but independent of *p*, and as such no breakpoints exist. Thus, for fixed *p*, a total order can be established over the set of all possible active sets.

### Definition 3.4

*(Dominant active sets and dominance breakpoints)* Let $\mathcal {A}^*(p)$ be the **dominant active set** (overall) at *p* if

4 $$\begin{aligned} \begin{aligned} \mathcal {A}^*(p)&= \mathop {\mathrm{arg\,max}}\limits _{\{\mathcal {A}_I,\mathcal {A}_D,\mathcal {A}_M\}} \left( f^*_{\mathcal {A}_I}(p), f^*_{\mathcal {A}_D}, f^*_{\mathcal {A}_M}(p)\right) \\&= \mathop {\mathrm{arg\,min}}\limits _{\{\mathcal {A}_I,\mathcal {A}_D,\mathcal {A}_M\}} \left( h^*_{\mathcal {A}_I}(p), h^*_{\mathcal {A}_D}, h^*_{\mathcal {A}_M}(p)\right) \end{aligned} \end{aligned}$$

and the optimal objective solution of Problem P–3.4 is $f^* = \max _p f^*_{\mathcal {A}^*(p)}(p)$, where:

- $\mathcal {A}_I$ is the **dominant input active set at** *p* if $\mathcal {A}_I(p) = \mathop {\mathrm{arg\,max}}\limits _{A\subseteq T_X}f^*_{A}(p)$.
- $\mathcal {A}_M$ is the **dominant mixed active set at** *p* if $\mathcal {A}_M(p)= \mathop {\mathrm{arg\,max}}\limits _{A\subseteq T_X\cup T_Z,\ A\setminus T_X\notin \{A,\emptyset \}}f^*_{A}(p)$.
- $\mathcal {A}_D$ is the **dominant direct active set** if $\mathcal {A}_D= \mathop {\mathrm{arg\,max}}\limits _{A\subseteq T_Z}f^*_{A}$.

A **dominance breakpoint** represents a *p* value where the dominant active set changes, i.e. $\forall \ 0<\epsilon <\epsilon _0$, where $\epsilon _0$ is a sufficiently small positive number,

5 $$\begin{aligned} \mathcal {A}^*(p-\epsilon ) \not = \mathcal {A}^*(p+\epsilon ) \text { and }\mathcal {A}^*(p-\epsilon ) \asymp _p \mathcal {A}^*(p+\epsilon ). \end{aligned}$$

*Input and mixed dominance breakpoints* are similarly defined as in Eq. (5) but with $\mathcal {A}^*$ replaced by $\mathcal {A}_I$ and $\mathcal {A}_M$, respectively. Let the sets of input and mixed dominance breakpoints be denoted by $\mathcal {B}_I$ and $\mathcal {B}_M$, respectively.

The input, direct and mixed active sets have different dominance properties and thus the analysis proceeds in Sects. 3.1–3.3 by active set type. Section 3.1 ignores directs and product quality constraints and focuses only on inputs. Since directs are ignored, the pool concentration *p* represents the output concentration, and hence *p* is assumed free of product quality bounds. The analysis of the *p*-parametric optimal objective $f^*(p)$ reveals a piecewise-linear structure associated with pairs of inputs acting as the dominant input active set. Section 3.2 treats the complementary case, ignoring inputs and focusing only on directs while assuming product quality constraints. Since inputs and therefore the pool are assumed to send no flow in this case, the optimal objective $f^*$ and the dominant direct active set are found independently of *p*. Finally, Sect. 3.3 integrates the Sects. 3.1–3.2 results, combining both inputs and directs under assumed product quality constraints to reveal a sparse, piecewise-monotone structure of the *p*-parametric optimal objective $f^*(p)$.

Sections 3.1–3.3 analytically and parametrically identify all (dominance) breakpoints, sparse dominant active sets and associated *p*-parametric solutions for Problem P–3.4. This analysis leads to a strongly-polynomial algorithm in Sect. 3.3 for solving the $\mathrm{I}{+}\mathrm{H}{-}1{-}1$ subclass formalized in Problem P–3.4. Furthermore the full structure of the *p*-parametric optimal objective function $f^*(p)$ developed in Sect. 3.3 is vital for Sects. 4–5.

### Remark 3.5

For any $i,j\in T_X\cup T_Z$ with $i\not =j,\ C_i=C_j$, if $\gamma _i\le \gamma _j$ precedence is given to the node *i* with cheaper flow, or at cost equality a random choice is made. After pre-filtering all feeds of equal concentrations on cost criteria, we are assured $\forall i, j\in T_X\cup T_Z, i\not =j$ that $C_i\not = C_j$. For any $i,j\in T_X\cup T_Z$ with $i\not =j,\ C_i < C_j$ if $\gamma _i=\gamma _j$ precedence is given to *i* if $C_i\in [P^L,P^U],\ C_j\notin [P^L,P^U]$ and vice versa. We apply the enumerated precedence rules throughout Sect. 3. This pre-filtering avoids undefined expressions, e.g. denominators with value zero in the subsequent sections.

### Inputs-only analysis (I${-}1{-}1$ sub-case)

This subsection considers the Problem P–3.4 restriction with no direct flows to the output ($z_i=0,\forall i \in T_Z$) and no product quality constraints on the pool concentration *p*. The resulting Problem P–3.1.5 is *p*-parametric, and thus we seek to find both the optimal solution and the full *p*-parametric structure. While Remark 3.1.1 observes the cardinality of the dominant input active set at any *p*, Proposition 3.1.5 explicitly identifies $\mathcal {A}_I(p)$. Theorem 3.1.6 then finds all input dominance breakpoints $\mathcal {B}_I$, the optimal solution and more importantly, the full piecewise-linear structure of the *p*-parametric optimal objective $f^*(p)$ motivated by Lemma 3.1.2. The piecewise structure is expanded in Sect. 3.3 in the presence of directs and product quality constraints, but remains fundamental to all analytical solutions found in the paper, including Sects. 4–5. 
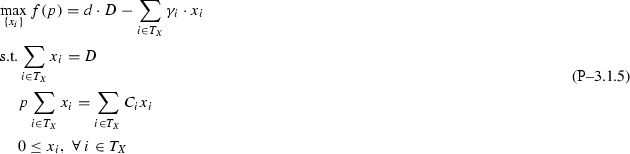

**Fig. 2:**
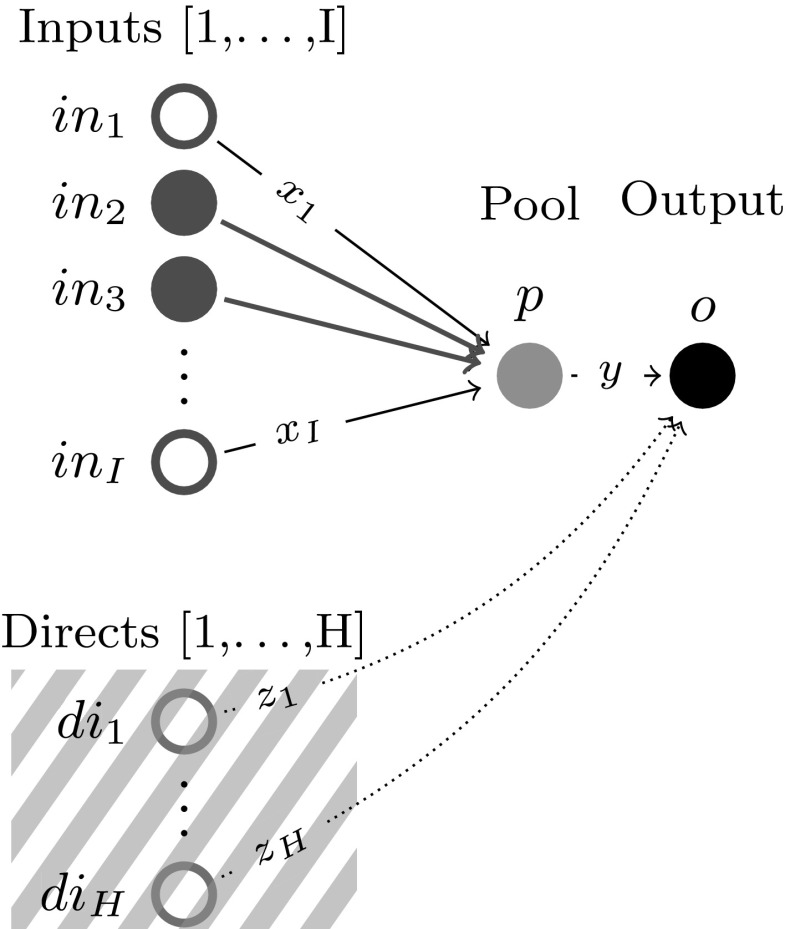
I-1-1 pooling network with directs ignored (hashed) and dominant pair of inputs (blue). (Color figure online)

#### Remark 3.1.1

(*Dominant input active set at p of cardinality* 2) For fixed *p*, Problem P–3.1.5 can be rewritten as a standard LP in the *x* variables with two active constraints at the optimal basis, implying the dominant input active set has cardinality 2, i.e. $|\mathcal {A}_I(p)| = 2$ (Fig. 2).

#### Lemma 3.1.2

(Linear *f*(*p*) and flows for active input pair) For $A = \{i,j\}$ and fixed *p* in Problem P–3.1.5, $f_A(p)$ is linear with respect to $p$ $:$

6 $$\begin{aligned} {\frac{\partial f_A}{\partial p}}= & {} -\frac{D(\gamma _i-\gamma _j)}{C_i-C_j}, \end{aligned}$$

7 $$\begin{aligned} \text {with flows} \quad x_i= & {} \frac{D(p-C_j)}{C_i-C_j},\quad x_j=\frac{D(p-C_i)}{C_j-C_i}. \end{aligned}$$

#### Proof

The flows in Eq. (7) result from $x_i+x_j=D,\ p=(C_ix_i+C_jx_j)/(x_i+x_j)$. Eq. (6) follows by substituting Eq. (7) flows into $f_A(p)=dD-\gamma _ix_i-\gamma _j x_j$ and differentiating w.r.t. *p*. $\square$

#### Definition 3.1.3

Input active pair $\{i,j\}$ can be viewed as one joint node denoted by $ij=\{i,j\}$ with the following flow to the pool and cost at pool concentration *p*, i.e.:

8 $$\begin{aligned} {\left\{ \begin{array}{ll} &{} x_{ij}(p)=x_i+x_j,\\ &{} \gamma _{ij}(p)= \left( \gamma _ix_{i}+\gamma _jx_j\right) \big / (x_i+x_j)=\big (\gamma _i(p-C_j)+\gamma _j(C_i-p)\big )\big /(C_i-C_j),\\ &{} p= \left( C_ix_{i}+C_jx_j\right) \big / (x_i+x_j),\\ \end{array}\right. }\quad \end{aligned}$$

where the second equality for $\gamma _{ij}(p)$ follows from Eq. (7). Note that $\gamma _{ij}(p)$ is a weighted average of $\gamma _i,\gamma _j$, uniquely determined at a fixed *p*, which in turn is a weighted average of $C_i,C_j$.

#### Proposition 3.1.4

(Domination condition between active input pairs) If $i,j,k,l\in T_X$, then $ij=\{i,j\} \succeq _p kl=\{k,l\}\Leftrightarrow$

9a $$\begin{aligned}&p\biggl (\frac{\gamma _i-\gamma _j}{C_i-C_j}\biggl )+ \biggl (\frac{\gamma _jC_i-\gamma _iC_j}{C_i-C_j}\biggl ) \le p\biggl (\frac{\gamma _k-\gamma _l}{C_k-C_l}\biggl )+ \biggl (\frac{\gamma _lC_k-\gamma _kC_l}{C_k-C_l}\biggl ) \qquad (p\text {-slope form}) \Leftrightarrow \end{aligned}$$

9b $$\begin{aligned}&\frac{\gamma _i(p-C_j)+\gamma _j(C_i-p)}{C_i-C_j}=\gamma _{ij}(p) \le \gamma _{kl}(p)=\frac{\gamma _k(p-C_l)+\gamma _l(C_k-p)}{C_k-C_l} \quad \text {(cost-based form)}.\nonumber \\ \end{aligned}$$

#### Proof

W.l.o.g. $C_i\ge p\ge C_j$ and $C_k\ge p\ge C_l$. Eq. (2) implies $ij\succeq _p kl\Leftrightarrow \gamma _ix_i+\gamma _jx_j\le \gamma _kx_k+\gamma _lx_l.$ Eq. (9b) follows from Definition 3.1.3 after substituting Eq. (7) for flows in the previous condition. Eq. (9a) follows from separating out the terms with factor/slope *p* in Eq. (9b). $\square$

#### Proposition 3.1.5

(Dominant input active set at *p*) For fixed *p*, if an input active pair $ij=\{i,j\}$ dominates at *p* any alternative pair, i.e.:

10 $$\begin{aligned} ij = \mathop {\mathrm{arg\,min}}\limits _{\{k,l\}\subseteq T_X} \gamma _{kl}(p) = \mathop {\mathrm{arg\,min}}\limits _{\{k,l\}\subseteq T_X} \frac{\gamma _k(p-C_l)+\gamma _l(C_k-p)}{C_k-C_l}, \quad \end{aligned}$$

then $\mathcal {A}_I(p)=ij$ for Problem P–3.1.5.

#### Proof

Follows from Remark 3.1.1 coupled with Proposition 3.1.4. $\square$

#### Theorem 3.1.6

(Inputs-only optimal solution and input dominance breakpoints)

(i) Input dominance breakpoints can occur only at input concentrations $C_i,\ i\in T_X$, hence,
  $$\begin{aligned} f^*=\max _{i\in T_X}f^*(C_i)=\max _{i\in T_X}D(d-\gamma _i), \end{aligned}$$
  which requires *I* (number of inputs) evaluations.
(ii) A full description of $f^*(p)$ can be obtained in strongly-polynomial time $O(I^3)$, with the set $\mathcal {B}_I$ of input dominance breakpoints,
  11a $$\begin{aligned} \mathcal {B}_I = \left\{ C_i \left| \ i\in T_X,\ \gamma _i< \mathop {\mathrm{arg\,min}}\limits _{\{k,l\}\subseteq T_X\setminus \{i\}} \gamma _{kl}(C_i)\right\} \right. . \end{aligned}$$
  Between any two consecutive elements of $\mathcal {B}_I$, the dominant input active set remains constant, i.e.
  11b $$\begin{aligned} C_i,C_j\in \mathcal {B}_I,\ \mathcal {B}_I\cap (C_i,C_j)=\emptyset \quad \Rightarrow \quad \mathcal {A}_I(p)=\{i,j\}\ \forall p\in [C_i,C_j] \end{aligned}$$

#### Proof

**Fig. 3:**
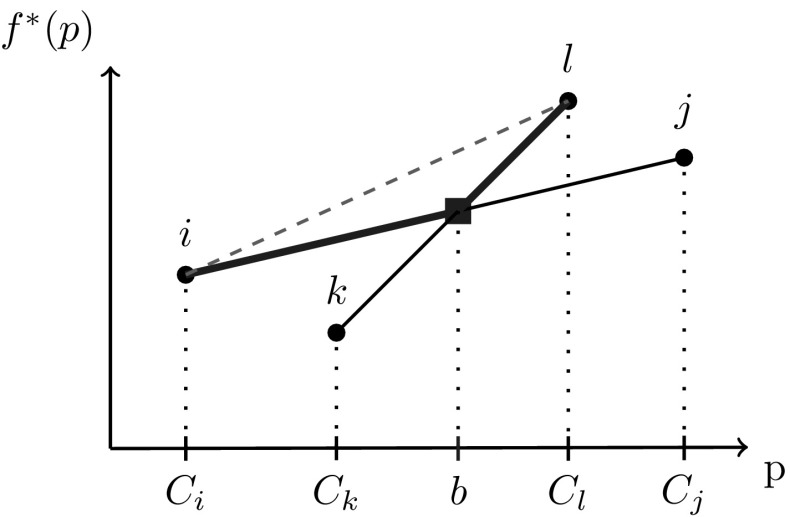
$i-j$ and $l-k$ lines represent $f^*(p)$ between individual node concentrations for pairs $\{i,\ j\}$ and $\{l,\ k\}$, respectively—*b* is the breakpoint concentration between the two node pairs. The bold blue lines show the optimal objective if *b* is a dominance breakpoint, and the green dashed line shows the optimal objective otherwise. (Color figure online)

(i) Since Proposition 3.1.5 implies $|\mathcal {A}_I(p)|=2$, let two such dominant active input pairs, $\{i,j\}$ and $\{k,l\}$, and w.l.o.g. assume $C_i<C_j$, $C_k<C_l$. Assume, to achieve a contradiction, that an input dominance breakpoint occurs at *b*, where $b\in (C_i,C_j)\cap (C_k,C_l)$. Consequently, again w.l.o.g. assume $\{k,l\}\succeq _p \{i,j\}$ $\forall p\in (b,C_l)$. As a result, in the geometric construction of Fig. 3, $i{-}l{-}j{-}k$ forms a quadrilateral with $f^*(b)$ at the intersection of its diagonals. Notice, $\forall p\in (C_i,C_l)$, the $f_{\{i,l\}}^*(p)$ values obtained on the side $i-l$ (dashed green) are higher than the optimal objective values obtained by going through the breakpoint *b* (lines in bold blue), contradiction. Therefore no input dominance breakpoint can occur at a pool concentration $b\notin \{C_i|\ i\in T_X \}$. Since Lemma 3.1.2 implies $f^*(p)$ is linear between any two input dominance breakpoints when an input pair is active, the assertion made follows.
(ii) To fully describe $f^*(p)$, if $C_i$ for fixed $i\in T_X$ is an input dominance breakpoint, then, according to Eq. (10), node *i* must strictly dominate at $C_i$ any input pair not containing it. Eq. (11b) follows via the definitions of $\mathcal {B}_I, \mathcal {A}_I$. $\square$

#### Remark 3.1.7

If product quality constraints are re-added to Problem P–3.1.5, then Theorem 3.1.6 still applies, with valid input dominance breakpoints $(\mathcal {B}_I \cap (P^L,P^U))\cup \{P^L,P^U\}$.

**Fig. 4:**
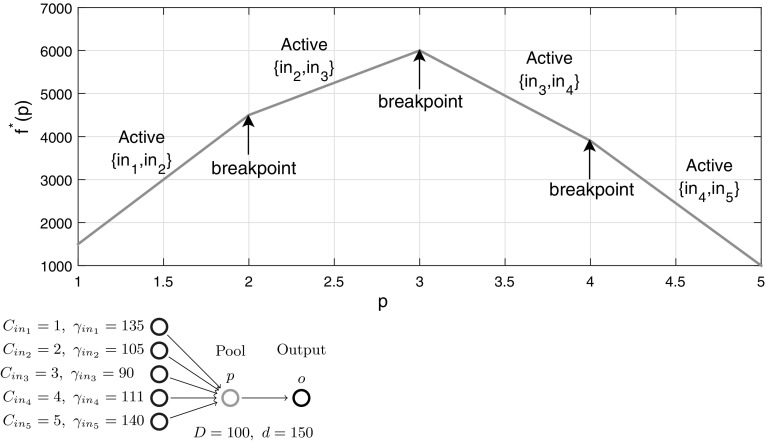
Optimal objective function $f^*(p)$ versus pool concentration *p* for a one pool, one output network with five inputs (parametrized with concentrations/costs). The objective is a piecewise-linear function of the pool concentration

We conclude the I${-}1{-}1$ sub-case analysis with a numerical example showcasing the implications of Theorem 3.1.6. For the Fig. 4 example with five inputs and no quality constraints, the function $f^*(p)$ reveals breakpoints at concentrations $C_2,\ C_3$ and $C_4$ in a piecewise-linear structure, as expected via Lemma 3.1.2. Furthermore, each *p*-interval between two breakpoints identifies the sparse dominant input active sets and their corresponding pair of active flow variables. The coupling of sparsity with piecewise-linear structure matches the Beale et al. [9] intuition. These special structures provide motivation to further explore *p*-parametric optimal objective structure on progressively more general problem subclasses in the remaining sections.

### Directs-only analysis (H${-}1{-}1$ sub-case)

This subsection restricts Problem P–3.4 to disallow flows from the input nodes to the pool node ($x_i=0,\forall i \in T_X$). The resulting Problem P–3.2.6, which is complementary to Sect. 3.1, also incorporates the product quality constraints. Optimal solutions implying direct-only flows are not parametric on *p*, since the problem is independent of pool concentration. This subsection therefore seeks the unique, dominant direct active set and its solution. Lemmas 3.2.2–3.2.3 give feasibility results. Given an active direct pair, Proposition 3.2.4 introduces the output concentration obtained at the optimum of Problem P–3.2.6. This enables an ordering among direct active pairs via Corollary 3.2.6 that together with the cardinality observation in Remark 3.2.1 leads to identifying the optimal solution and the dominant direct active set in Theorem 3.2.7. The scope of this subsection goes beyond identifying the LP solution of Problem P–3.2.6 as the results developed herein are central to the more involved analysis in Sect. 3.3. 
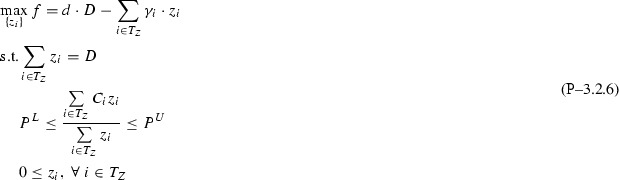

**Fig. 5:**
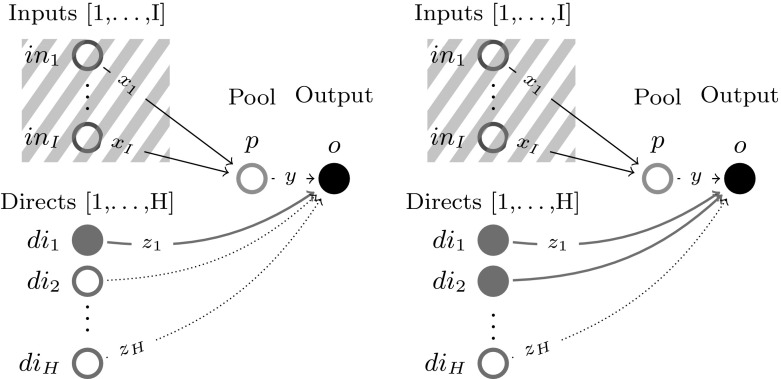
H${-}1{-}1$ pooling network with inputs ignored (hashed) and the dominant pair/single direct active set (filled green, Theorem 3.2.7). (Color figure online)

#### Remark 3.2.1

*(Dominant direct active set of maximum cardinality* 2*)* Problem P–3.2.6 can be rewritten as a standard LP in the *z* variables with at most two active constraints at the optimal basis, implying the dominant direct active set has at most cardinality 2, i.e. $|\mathcal {A}_D| \le 2$ (Fig. 5).

#### Lemma 3.2.2

(Simple feasibility conditions)

(i) A feasible direct active set (solution) exists $\Leftrightarrow \exists i\in T_Z$ s.t. $P^L \le C_i \le P^U$ or $\exists i,j\in T_Z$ s.t. $C_i<P^L, C_j > P^U$.
(i) A direct active pair {i, j} is feasible $\Leftrightarrow$ *i* and *j* are feasible or $(P^U-C_i)(P^U-C_j)<0$ or $(P^L-C_i)(P^L-C_j)<0$.

#### Proof

(i) If $\exists i\in T_Z$ s.t. $P^L \le C_i \le P^U$ then Problem P–3.2.6 is obviously feasible. Alternatively, w.l.o.g., if $C_i<P^L$, there must $\exists j\in T_Z$ with $C_j > P^L$ so an output concentration within $[P^L,P^U]$ can be obtained. However, this case implicitly assumed $\not \exists i\in T_Z$ s.t. $P^L \le C_i \le P^U$, and consequently $C_j > P^U$, concluding the proof.

(ii) Both $(P^U-C_i)(P^U-C_j)<0$ or $(P^L-C_i)(P^L-C_j)<0$ imply $C_i$ and $C_j$ are on opposite sides of a product quality bound and thus the linear combination of their concentrations implied by active $\{i,j\}$ can be within $[P^L,P^U]$, making $\{i,j\}$ feasible. $\square$

#### Lemma 3.2.3

(Pair dominating both its individual nodes and feasibility) If $\{i,j\}$ is a feasible direct active pair, then:

$$\begin{aligned} \{i,j\}\succeq i,j \Leftrightarrow \alpha =\mathop {\mathrm{arg\,min}}\limits _{i,j}(\gamma _i,\gamma _j) \text { is infeasible, i.e. } \, C_\alpha \not \in [P^L,P^U]. \end{aligned}$$

#### Proof

’$\Rightarrow$’: If both $C_i,C_j\in [P^L,P^U]$, then ${{\mathrm{arg\,min}}}_{i,j}(\gamma _i,\gamma _j) \succeq \{i,j\}$, contradiction, so we can assume w.l.o.g. $C_i\not \in [P^L,P^U]$. If also $C_j\not \in [P^L,P^U]$ both *i* and *j* are infeasible alone, but $\{i,j\}$ can be feasible if the second condition in Lemma 3.2.2.i is met. In the previous case, one of the nodes *i*, *j* has the properties needed. Else if $C_j\in [P^L,P^U]$ and $\gamma _i>\gamma _j$, then $j\succeq \{i,j\}$ - therefore $\gamma _i<\gamma _j$. The reverse proof ’$\Leftarrow$’ is trivial. $\square$

#### Proposition 3.2.4

(Optimal output concentration for feasible direct pairs) Given feasible direct active pair $\{i,j\}$ with $\{i,j\}\succeq i,j$, define *P*(*i*, *j*), based on properties of *i*, *j*, as

12 $$\begin{aligned} \begin{aligned} P(i,j)&= \left( \frac{P^U+P^L}{2}\right) - \left( \frac{P^U-P^L}{2} \right) \cdot \ \mathop {\mathrm {sgn}}\left( (C_i-C_j)(\gamma _i-\gamma _j)\right) \\&= {\left\{ \begin{array}{ll} P^L,\text { if } (C_i-C_j)(\gamma _i-\gamma _j)>0\\ P^U,\text { if } (C_i-C_j)(\gamma _i-\gamma _j)<0. \end{array}\right. } \end{aligned} \end{aligned}$$

Then, *P*(*i*, *j*) represents the output concentration at optimality, reducing Problem P–3.2.6 to 
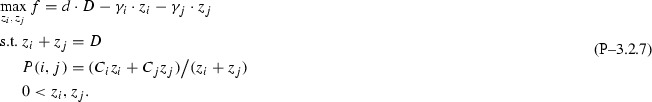


#### Proof

Proof in “Appendix A”. Note that due to Remark 3.5 and Lemmas 3.2.2–3.2.3 we have $(C_i-C_j)(\gamma _i-\gamma _j)\not =0$. $\square$

The result implies that for a feasible direct active pair $\{i,j\}$ with $\{i,j\}\succeq i,j$, Problem P–3.2.7 is analogous to the input-only Problem P–3.1.5, with input flows $x_i$ replaced by direct flows $z_i$ and fixed pool concentration *p* replaced by a product quality limit *P*(*i*, *j*) (either lower or upper). Thus, the flow and dominance results for pairs in Sect. 3.1 are mirrored via Corollaries 3.2.5–3.2.6. Moreover, any pair viable as the dominant direct active set needs to first dominate both its individual nodes, so only such pairs and their solutions are of interest.

#### Corollary 3.2.5

The flows of a feasible direct active pair $\{i,j\}$ in Problem P–3.2.7 are:

13 $$\begin{aligned} z_i=\frac{D(P(i,j)-C_j)}{C_i-C_j},\quad z_j=\frac{D(P(i,j)-C_i)}{C_j-C_i}. \end{aligned}$$

#### Proof

Analogous to Lemma 3.1.2, but for Problem P–3.2.7 rather than Problem P–3.1.5. $\square$

#### Corollary 3.2.6

(Domination condition between active direct pairs)

For $i,j,k,l\in T_Z$ with $\{i,j\},\{k,l\}$ feasible, $ij=\{i,j\} \succ kl=\{k,l\}\Leftrightarrow$

14a $$\begin{aligned}&P(i,j)\biggl (\frac{\gamma _i-\gamma _j}{C_i-C_j}\biggl )+\frac{\gamma _jC_i-\gamma _iC_j}{C_i-C_j} < P(k,l)\biggl (\frac{\gamma _k-\gamma _l}{C_k-C_l}\biggl )+\frac{\gamma _lC_k-\gamma _kC_l}{C_k-C_l}\Leftrightarrow \end{aligned}$$

14b $$\begin{aligned}&\frac{\gamma _i(P(i,j)-C_j)+\gamma _j(C_i-P(i,j))}{C_i-C_j}=\gamma _{ij} <\gamma _{kl} =\frac{\gamma _k(P(k,l)-C_l)+\gamma _l(C_k-P(k,l))}{C_k-C_l}.\qquad \qquad \nonumber \\ \end{aligned}$$

#### Proof

Analogous to Proposition 3.1.4, but for Problem P–3.2.7 rather than Problem P–3.1.5. $\square$

#### Theorem 3.2.7

*(Directs-only optimal solution and dominant direct active set)*

Given $\{i,j\}$ a feasible direct active pair that dominates any such alternative pairs, i.e.:

15 $$\begin{aligned} \{i,j\} = \mathop {\mathrm{arg\,min}}\limits _{\begin{array}{c} \text {feasible } \{k,l\}\subseteq T_Z,\\ \{k,l\}\succeq k,l \end{array}} \gamma _{kl} = \mathop {\mathrm{arg\,min}}\limits _{\begin{array}{c} \text {feasible } \{k,l\}\subseteq T_Z,\\ \{k,l\} \succeq k,l \end{array}} \frac{\gamma _k(P(k,l)-C_l)+\gamma _l(C_k-P(k,l))}{C_k-C_l}, \end{aligned}$$

which can be found in strongly-polynomial time $O(H^2)$, then either:

- If $\{i,j\}\succeq i,j$ then $\mathcal {A}_D=\{i,j\}$ with the flows in Eq. (13) and $f^*=D\cdot (d-\gamma _{ij})$.
- Else, $\mathcal {A}_D= \alpha = {{\mathrm{arg\,min}}}_{\alpha \in \{i,j\}} \gamma _\alpha$ with $z_{\alpha }=D$ and $f^*=D\cdot (d-\gamma _{\alpha })$.

#### Proof

Eq. (15) follows directly from Corollary 3.2.6.

- If $\{i,j\}\succeq i,j$, Lemma 3.2.3 implies one of the nodes, w.l.o.g *j*, is infeasible, so w.l.o.g $C_j>P^U,C_i<P^U$ and $\gamma _j<\gamma _i$. If $\exists k\in T_Z\setminus \{i\}$ s.t. $C_k<P^U,\ \gamma _k<\gamma _i$, then $\{k,j\}\succeq \{i,j\}$, contradiction. Therefore (feasible) *i* dominates any other alternative feasible direct, and by transitivity, $\{i,j\}\succeq i\succeq k\ \forall k\in T_Z\setminus \{i\}, C_k\in [P^L,P^U]$ . The latter and Remark 3.2.1 imply that $\mathcal {A}_D=\{i,j\}$.
- Assume $i\succeq \{i,j\}$, so *i* is feasible with $P^L \le C_i \le P^U$. If $\gamma _j<\gamma _i$ then $\{i,j\}$ as a pair with a linearly weighted cost would dominate *i*, contradiction, and therefore $\gamma _j>\gamma _i$. If more restrictively, $P^L< C_i < P^U$, since by transitivity $i\succeq \{i,j\}\succeq \{i,k\}\ \forall k\in T_Z\setminus \{i,j\}$ ($\{i,k\}$ feasible due to $P^L< C_i < P^U$), therefore $\gamma _k>\gamma _i \ \forall k\in T_Z\setminus \{i\}$ and $\mathcal {A}_D=i$. Now the complementary restriction of $C_i=P^U$ is assumed ($C_i=P^L$ is analogous). If $\exists k\in T_Z\setminus \{j\},\ C_k<P^U,\ \gamma _k<\gamma _i$ then $\{i,k\}\succeq i\succeq \{i,j\}$, contradiction, therefore $\forall k\in T_Z\setminus \{j\},\ C_k<P^U$ we have $\gamma _k>\gamma _i$. Additionally, since $\{i,j\}$ feasible implies $C_j<P^U$, if $\exists k\in T_Z$ s.t. $C_k>P^U, \gamma _k<\gamma _i$ then $\{k,j\}\succeq \{i,j\}$, contradiction, thus $\forall k\in T_Z,\ C_k>P^U,\ \gamma _k>\gamma _i$. Therefore, all sub-cases after assuming $i\succeq \{i,j\}$ result in $\mathcal {A}_D=i$.$\square$

### Inputs and directs analysis (I${+}\mathrm{H}{-}1{-}1$ subclass)

This subsection considers the original *p*-parametric Problem P–3.4, allowing mixed active sets of both input and direct nodes. Theorem 3.3.1 uses the interplay of earlier results for both input (Sect. 3.1) and direct (Sect. 3.2) active sets to pinpoint mixed active sets that can be dominant (overall) active sets as triples of two inputs and one direct. This section focuses on mixed triples not dominated by the dominant input active set $\mathcal {A}_I$. Definition 3.3.2 first extends the feasibility conditions from Sect. 3.2 for mixed triples viewed as direct pairs to *p*-intervals by partitioning any *p*-interval ${\varPhi }$ around $\{P^L,P^U\}$ if necessary and building $Q({\varPhi })$, the set of directs making the mixed active set feasible. Definition 3.3.2e also extends the directs domination result in Lemma 3.2.3 to *p*-intervals. Lastly, Definition 3.3.2f splits *p*-intervals ${\varPhi }$ into sub-intervals ${\varPhi }_I$ and ${\varPhi }_M$ based on whether the dominant mixed active triple is dominated or not by $\mathcal {A}_I$ over the sub-intervals, respectively. Using the breakpoints between mixed and input active sets identified in Lemma 3.3.3, Lemma 3.3.4 then implements the ${\varPhi }_I/{\varPhi }_M$ split of any interval ${\varPhi }$.

Based on the latter results, Proposition 3.3.5 finds the dominant mixed active set for fixed *p* and all mixed dominance breakpoints, while Proposition 3.3.6 finds all dominance breakpoints. Moreover, Proposition 3.3.7 finds the *p*-parametric optimal objective function to be monotone convex/concave for mixed active sets. Consequently, Theorem 3.3.8 summarizes all cases of optimal objective monotonicity. Finally, Theorem 3.3.9 uses all objective monotonicity results to find the optimal solution at a breakpoint dominance point in strongly-polynomial time.

#### Theorem 3.3.1

*(Dominant mixed active set that can dominate overall at fixed* *p* *and its flows)* For fixed *p* and $i,j\in T_X$, $\mathcal {A}_I(p)=\{i,j\}=ij$ (as in Sect. 3.1, with no product quality constraints) $\Rightarrow$ $\mathcal {A}_M(p)=\{i,j,q\},\ q\in T_Z,$ with flow solutions,

16 $$\begin{aligned} \begin{aligned}x_i&=\frac{D(p-C_j)(P(ij,q)-C_q)}{(C_i-C_j)(p-C_q)},\ x_j=\frac{D(p-C_i)(P(ij,q)-C_q)}{(C_j-C_i)(p-C_q)}, \\ z_q&=\frac{D(p-P(ij,q))}{(p-C_q)}, \end{aligned} \end{aligned}$$

or else $$\mathcal {A}_M(p) \preceq _p \mathcal {A}_I(p)$$.

#### Proof

Fixing *p*, the concentration the active input set delivers via the pool towards the output concentration, implies product quality constraints on output concentration become irrelevant when considering only the active inputs part of a mixed active set. This observation allows to first pre-solve the inputs-only sub-Problem P–3.3.8, where the objective function $f_A(p, x_A(p))$ is now also parametric on variable total input flow $x_A(p)$ for an active input set *A*. According to Proposition 3.1.5, $A = ij = \{i,j\}$ at optimality for Problem P–3.3.8, with the solution in Eq. (18).
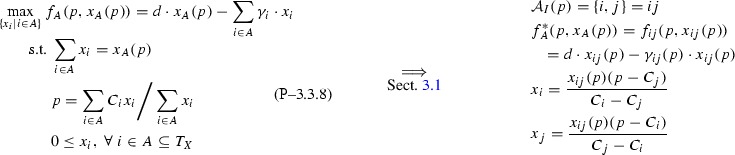


Now the optimal input-only parametric solution in Eq. (18) can be incorporated into the full Problem P–3.4. This results in a direct-only Problem P–3.3.9 that is augmented by the active joint input node *ij* acting via the pool as an active (additional) direct node with fixed concentration *p* and variable flow $x_{ij}(p)$. Denote the dominant direct active set for the augmented Problem P–3.3.9 at fixed *p* with $\mathcal {A}_D^p$ and note that Problem P–3.3.9 accounts for any possible dominant mixed active set $\mathcal {A}_M(p)$. Applying Theorem 3.2.7 to Problem P–3.3.9 and taking into account *ij* must be active implies that, given $\{ij,q\}\succeq _p \{ij,l\}\ \forall l\in T_Z,\ \{ij,l\}$ feasible and *p* fixed, if $\{ij,q\}\succeq _p ij$ then $\mathcal {A}_D^p=\{ij,q\}=\mathcal {A}_M(p)$ or else $\mathcal {A}_D^p=ij=\mathcal {A}_I(p)\succeq _p \mathcal {A}_M(p)$. Therefore, a mixed active set can be the overall dominant active set if $\mathcal {A}_D^p=\{ij,q\}=\mathcal {A}_M(p)$ and Eq. (20a) is the solution to Problem P–3.3.9.
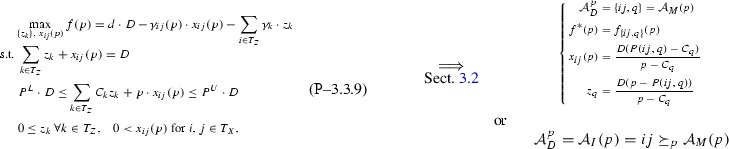


Finally, the flow solutions in Eq. (16) are found by combining Eq. (18) with Eq. (20a). $\square$

#### Definition 3.3.2

*(Feasibility/domination extensions to* *p*-*intervals around quality bounds)*

- Let sub-intervals of the partition $\{P^L,P^U\}$ of inputs/directs concentrations be denoted by:
  $$\begin{aligned} \begin{aligned} I^X_{L}&= \big [\min \limits _{i\in T_X}C_i, P^L\big ),\\ I^Z_{L}&= \big [\min \limits _{i\in T_Z}C_i, P^L\big ), \end{aligned}\quad I_{LU}=\big [P^L,P^U\big ],\quad \begin{aligned} I^X_{U}&= \big (P^U, \max \limits _{i\in T_X}C_i \big ],\\ I^Z_{U}&= \big (P^U, \max \limits _{i\in T_Z}C_i \big ]. \end{aligned} \end{aligned}$$
- Let ${\varPhi }\subseteq [C_i,C_j]$ denote a closed *p*-interval between two consecutive input dominance breakpoints, i.e. $C_i,C_j\in \mathcal {B}_I,\ \mathcal {B}_I\cap (C_i,C_j)=\emptyset$, with $(\forall p\in {\varPhi })\ \mathcal {A}_I(p)=ij$. Let ${\varPhi }\in \{[C_i,C_j]\cap I_L^X,\ [C_i,C_j]\cap I_{LU},\ [C_i,C_j]\cap I_U^X \}$ such that:
  $$\begin{aligned} \mathbb {1}_{{\varPhi }\subseteq I^X_L}+\mathbb {1}_{{\varPhi }\subseteq I^X_{LU}}+\mathbb {1}_{{\varPhi }\subseteq I^X_{U}}=1. \end{aligned}$$
- Let $Q({\varPhi })$ be the set of directs with concentration outside ${\varPhi }$’s partition around $\{P^L,P^U\}$, i.e.:
  $$\begin{aligned} \begin{aligned} Q({\varPhi })&= \big \{q\in T_Z \big |\ C_q\in [\min \limits _{i\in T_Z}C_i,\max \limits _{i\in T_Z}C_i]\setminus I^Z_\beta , \text { where } {\varPhi }\subseteq I^X_\beta ,\ \beta \in \{L,LU,U\} \big \}\\&= \big \{q\in T_Z \big |\ \text { mixed active set }\{ij,q\} \text { is feasible, where}\ (\forall p\in {\varPhi })\ \mathcal {A}_I(p)=ij \big \}. \end{aligned} \end{aligned}$$
- Let $R({\varPhi })\subseteq Q({\varPhi })$ denote a subset s.t. $(\forall q\in R({\varPhi }))(\forall p\in {\varPhi })\ \gamma _q < \gamma _{ij}(p)$. Since $\gamma _{ij}(p)$, as defined in Eq. (8), is a linear function of *p* with extremes at ${\varPhi }$ endpoints,
  $$\begin{aligned} \begin{aligned} R({\varPhi })=\big \{q\in Q({\varPhi }) \big |\ \gamma _q<\max \{\gamma _{ij}(b_l),\gamma _{ij}(b_u)\}, \text { where } {\varPhi }=[b_l,b_u]\big \}. \end{aligned} \end{aligned}$$
- Let $$\begin{aligned} {\varTheta }({\varPhi }) = {\left\{ \begin{array}{ll} R({\varPhi })\text { if } R({\varPhi })\not =\emptyset \text { or }\ \ {\varPhi }\subseteq I_{LU},\\ Q({\varPhi })\text { if } R({\varPhi }) =\emptyset \text { and } {\varPhi }\not \subseteq I_{LU}. \end{array}\right. } \end{aligned}$$
- Let ${\varPhi }_I,{\varPhi }_M$ be partition sub-intervals of ${\varPhi }$, where $\mathcal {A}_I, \mathcal {A}_M$ dominate, respectively. Assuming $(\forall p\in {\varPhi })\ \mathcal {A}_I(p)=ij$,
  21 $$\begin{aligned} \begin{aligned}&{\varPhi }_I = \left\{ p\in {\varPhi }|\ (\forall q\in {\varTheta }({\varPhi }))\ \gamma _{ij}(p)\le \gamma _{q} \Rightarrow \text {if }{\varPhi }\subseteq I_{LU}\text { then } (\forall q)\ ij\succeq _p \{ij,q\}, \mathcal {A}_I(p)\succeq _p \mathcal {A}_M(p) \right\} ,\\&{\varPhi }_M=\{ p\in {\varPhi }|\ (\exists q\in {\varTheta }({\varPhi }))\ \gamma _{ij}(p)\ge \gamma _{q} \Leftrightarrow (\exists q\in {\varTheta }({\varPhi }))\ ij\preceq _p \{ij,q\} \Leftrightarrow \mathcal {A}_I(p)\preceq _p \mathcal {A}_M(p) \}.\\ \end{aligned} \end{aligned}$$

Thus, ${\varTheta }({\varPhi })$ extends Lemmas 3.2.2 and 3.2.3 from fixed *p* to interval ${\varPhi }$ (see proof of Lemma 3.3.4.i).

#### Lemma 3.3.3

(Domination/breakpoints between dominant input and mixed active sets)

If $$i,j\in T_X,\ q\in T_Z$$, then $$ij=\{i,j\}\preceq _p\{ij,q\}=\{i,j,q\} \ \Leftrightarrow $$

22 $$\begin{aligned} \gamma _{ij}(p)\ge \gamma _q\ \Leftrightarrow \ \gamma _{ij}(p)\ge \gamma _{\{ij,q\}}(p):=\frac{\gamma _q(P(ij,q)-p)+\gamma _{ij}(p)(C_q-P(ij,q))}{C_q-p},\qquad \end{aligned}$$

with $\{i,j\}\asymp _p\{i,j,q\}$ at breakpoint pool concentration $p= \frac{C_i(\gamma _q-\gamma _j)-C_j(\gamma _q-\gamma _i)}{\gamma _i-\gamma _j}.$

#### Proof

Proof in “Appendix A”. $\square$

#### Lemma 3.3.4

For a given *p*-interval ${\varPhi }=[b_l,b_u]$, where $b_l,b_u \in \mathcal {B}_I\cup \{C_i,C_j\}$, $(\forall p\in {\varPhi })\ \mathcal {A}_I(p)=ij$, we have:

(i) $$(\forall p\in {\varPhi })\ (\mathcal {A}_M(p)=\{ij,q\} \Rightarrow q\in {\varTheta }({\varPhi }))$$.
(ii) $(\forall {\varPhi })$ Sub-intervals ${\varPhi }_I,{\varPhi }_M$ can be found explicitly as:
  23 $$\begin{aligned} \left\{ \begin{aligned}&(a)\ {\varPhi }_I={\varPhi },\ {\varPhi }_M=\emptyset , \text { if } {\varTheta }({\varPhi })=\emptyset ,\\&(b)\ {\varPhi }_I=\{ p\in {\varPhi }|\ b_l \le p \le \min (S) \},\ {\varPhi }_M=\{ p\in {\varPhi }|\ \min (S) \le p \le b_u \}, \text { else if } \frac{\gamma _i-\gamma _j}{C_i-C_j}>0,\\&(c)\ {\varPhi }_I=\{ p\in {\varPhi }| \max (S) \le p \le b_u \},\ {\varPhi }_M=\{ p\in {\varPhi }|\ b_l \le p \le \max (S) \}, \text { else if } \frac{\gamma _i-\gamma _j}{C_i-C_j}<0, \end{aligned}\right. \end{aligned}$$
  where:
  24 $$\begin{aligned} \begin{aligned} S&=\{p|\ \{i,j\}\asymp _p\{i,j,q\},\ \forall q\in {\varTheta }({\varPhi }) \} \\&= \left\{ \frac{C_i(\gamma _q-\gamma _j)-C_j(\gamma _q-\gamma _i)}{\gamma _i-\gamma _j}\bigg |\ \forall q\in {\varTheta }({\varPhi }) \right\} . \end{aligned} \end{aligned}$$
(iii) $(\forall q\in {\varTheta }({\varPhi })) (\forall p\in \hat{{\varPhi }}\in \{{\varPhi }_I,{\varPhi }_M\} )$ if $\mathcal {A}_M(p)=\{ij,q\}$ then *P*(*ij*, *q*) reduces to:
  25 $$\begin{aligned} P(ij,q)={\left\{ \begin{array}{ll} P^L,\text {if } ((C_q<b_u)\oplus (\hat{{\varPhi }}={\varPhi }_M))\wedge (P^L-C_q)(P^L-b_u)<0,\\ P^U,\text {if } ((C_q>b_u)\oplus (\hat{{\varPhi }}={\varPhi }_M))\wedge (P^U-C_q)(P^U-b_u)<0, \end{array}\right. } \end{aligned}$$
  independent of specific $p\in \hat{{\varPhi }}$.

#### Proof

(i) The restriction $Q({\varPhi })\in T_Z$, and its subset $R({\varPhi })\in T_Z$, enforces Lemma 3.2.2 feasibility for $\{ij,q\},\ \forall p\in {\varPhi }$ viewed as a direct active set. Set ${\varTheta }({\varPhi })$ also enforces the Lemma 3.2.3 domination condition $\{ij,q\}\succeq _p ij \Leftrightarrow \forall p\in {\varPhi }\ \gamma _q\le \gamma _{ij}(p)$ via ${\varTheta }({\varPhi })=R({\varPhi })$. When $R({\varPhi })=\emptyset$ and ${\varPhi }\subseteq I_{LU}$ then *ij* feasible and $(\forall q\in Q({\varPhi }))(\forall p\in {\varPhi })\ \gamma _q\ge \gamma _{ij}(p) \Leftrightarrow (\forall p\in {\varPhi })\ ij\succeq \mathcal {A}_M(p)$, in which case ${\varTheta }({\varPhi })=R({\varPhi })=\emptyset$ since $(\forall p\in {\varPhi })\ \mathcal {A}_M(p)\not =\mathcal {A}^*(p)$. When $R({\varPhi })=\emptyset$ and ${\varPhi }\not \subseteq I_{LU}$ then *ij* infeasible, and since a direct needs to be active for feasibility, in this case ${\varTheta }({\varPhi })=Q({\varPhi })$ and $(\forall q\in Q({\varPhi }))(\forall p\in {\varPhi })\ \gamma _q\ge \gamma _{ij}(p)$ but $\{ij,q\}\succeq _p ij,q$. Therefore in this case, ${\varTheta }({\varPhi })$ is extended from $R({\varPhi })$ to $Q({\varPhi })$.

(ii) Case (a) results by construction of ${\varPhi }_I,{\varPhi }_M, {\varTheta }({\varPhi })$. For Case (b)-(c), *S* is built as the set of all breakpoints from Lemma 3.3.3 where $\gamma _{ij}(p)=\gamma _{q},\forall p\in S, q\in {\varTheta }({\varPhi })$. From Eq. (8), $\partial \gamma _{ij}(p)/\partial p = (\gamma _i-\gamma _j)/(C_i-C_j)$, which implies if $\partial \gamma _{ij}(p)/\partial p>0$ then $\gamma _{ij}(p)$ is increasing with *p*. Therefore, given $\forall b\in S,\gamma _{ij}(b)=\gamma _q$, if $\partial \gamma _{ij}(p)/\partial p>0$ then $\forall p\le b\ \gamma _{ij}(p)\le \gamma _q$ and vice-versa. Consequently, if $\partial \gamma _{ij}(p)/\partial p>0$ then ${\varPhi }_I$ is the restriction of ${\varPhi }$ up till $\min (S)$, and otherwise ${\varPhi }_I$ is the restriction of ${\varPhi }$ from $\max (S)$.

Consequently, $\forall p\in {\varPhi }_I$, if *ij* is feasible (${\varPhi }\subseteq I_{LU}$) then *ij* dominates any mixed active set $\{ij,q\}$ and Theorem 3.3.1 then implies $(\forall p\in {\varPhi }_I)\ \mathcal {A}_I(p)\succeq _p \mathcal {A}_M(p)$. Complementary, $\{ij,q\}$ is feasible by construction ($q\in {\varTheta }({\varPhi })$), and therefore $\forall p\in {\varPhi }_M\ \exists q\in {\varTheta }({\varPhi })\ \gamma _{ij}(p)\ge \gamma _{q} \Leftrightarrow \exists q\in {\varTheta }({\varPhi })\ ij\preceq _p \{ij,q\}\Leftrightarrow \mathcal {A}_I(p)\preceq _p \mathcal {A}_M(p)$.

(iii) Assume first $\hat{{\varPhi }}={\varPhi }_I\not ={\varPhi }_M$. Then, Eq. (21) implies $(\forall q\in {\varTheta }({\varPhi }))(\forall p \in \hat{{\varPhi }})\ \gamma _{ij}(p)\le \gamma _q$. Consequently, the expression for *P*(*ij*, *q*) according to Eq. (12) reduces to Eq. (25). Now assume $\hat{{\varPhi }}={\varPhi }_M\not ={\varPhi }_I$. Then, Eq. (21) implies $(\forall p\in \hat{{\varPhi }})\ \mathcal {A}_M(p)=\{ij,q\}\succeq _p \mathcal {A}_I(p)$ and hence $(\forall p\in \hat{{\varPhi }})\ \gamma _{ij}(p)\ge \gamma _{q}$. Thus, again, the expression for *P*(*ij*, *q*) reduces to Eq. (25). For both possible $\hat{{\varPhi }}\in \{{\varPhi }_I,{\varPhi }_M\}$, due to the construction of ${\varPhi }$ and ${\varTheta }({\varPhi })$ with $q\in {\varTheta }({\varPhi })$, $(\forall p\in \hat{{\varPhi }})\ (C_q<p)=(C_q<b_u)$, and thus the comparison becomes independent of a specific $p\in \hat{{\varPhi }}$. Furthermore, to ensure feasibility of $\{ij,q\}$ according to Lemma 3.2.2.ii, $(P(ij,q)-C_q)(P(ij,q)-b_u)<0$ is enforced explicitly in Eq. (25). $\square$

#### Proposition 3.3.5

For $$\hat{{\varPhi }}=[b_l,b_u]\in \{{\varPhi }_M;\ {\varPhi }_I \not \subseteq I_{LU}\}\subseteq {\varPhi },\ (\forall p\in {\varPhi })\ \mathcal {A}_I(p)=ij$$, $$Q({\varPhi })\not =\emptyset $$:

(i) *(The dominant mixed active set at* *p)* For fixed $p\in \hat{{\varPhi }},\ \mathcal {A}_M(p)=\{ij,q\}\succeq _p \mathcal {A}_I(p)$ with
  26 $$\begin{aligned} \ q = \mathop {\mathrm{arg\,min}}\limits _{r\in {\varTheta }({\varPhi })} \left( \gamma _{\{ij,r\}}(p) := {\frac{\gamma _r(P(ij,r)-p)+\gamma _{ij}(p)(C_r-P(ij,r))}{C_r-p}}\right) . \end{aligned}$$
(ii) *(The set of mixed dominance breakpoints over* $\hat{{\varPhi }}$*)*
  27 $$\begin{aligned} \begin{aligned} \mathcal {B}_M&\supseteq \left\{ p\in {{\mathrm{int}}}{\hat{{\varPhi }}}|\ \{ij,q\}\asymp _{p}\{ij,r\} \wedge q,r\in \mathcal {A}_M(p),\ \forall q,r\in {\varTheta }({\varPhi }) \right\} \\&= \left\{ p\in {{\mathrm{int}}}{\hat{{\varPhi }}}|\ p\in \text {SqrRoots}\left( {\varGamma }\big (p,P(ij,q),P(ij,r)\big )\right) \wedge q,r\in \mathcal {A}_M(p),\ \forall q,r\in {\varTheta }({\varPhi }) \right\} , \end{aligned} \end{aligned}$$
  with $(\forall q\in {\varTheta }({\varPhi }))\ P(ij,q)$ as in Eq. (25) and independent of *p*; $b_l,b_u\in \mathcal {B}_M$ as well.

#### Proof

(i) If $\hat{{\varPhi }}\not \subseteq I_{LU}$, then $\mathcal {A}_I(p)=ij$ is infeasible and by default $\mathcal {A}_M(p)\succeq _p \mathcal {A}_I(p)$. If $\hat{{\varPhi }}={\varPhi }_M$, then, from the construction of ${\varPhi }_M$ in Eq. (21), $\mathcal {A}_M(p)\succeq _p \mathcal {A}_I(p)$. Thus Theorem 3.3.1 asserts via Eq. (20a) that $\mathcal {A}_D^p =\{ij,q\}=\mathcal {A}_M(p)$ for a $q\in {\varTheta }({\varPhi })$, as required by Lemma 3.3.4.i. By viewing $\{ij,q\}$ as a dominant direct pair with *ij* active in order to find $\mathcal {A}_D^p$, the condition Eq. (15) in Theorem 3.2.7 becomes Eq. (26). Since *p* is fixed, Eq. (26) can be solved using the original Eq. (12) for $P(ij,q)\ \forall q\in {\varTheta }({\varPhi })$.

(ii) “Appendix A” proves Eq. (27) and introduces function ${\varGamma }\big (p,P(ij,q),P(ij,r))$ which is quadratic in *p* and linear in *P*(*ij*, *q*), *P*(*ij*, *r*). To solve the function ${\varGamma }\big (p,P(ij,q),P(ij,r)\big )$ as a quadratic of *p*, $(\forall q\in {\varTheta }({\varPhi }))\ P(ij,q)$ has to be independent of *p* via the form in Eq. (25). First, Lemma 3.3.4.iii implies $\forall p\in \hat{{\varPhi }}$ that if $\{ij,q\}=\mathcal {A}_M(p)$ then *P*(*ij*, *q*) in Eq. (25) is correct; therefore any dominant breakpoint within ${\varPhi }$ between $\{ij,q\}$ and another dominant mixed set is captured in $\mathcal {B}_M$. Second, Lemma 3.3.4.iii implies if Eq. (25)$\not \Leftrightarrow$Eq. (12) for $P(ij,q),\ q\in {\varTheta }({\varPhi }),$ then feasible $\{ij,q\}\not =\mathcal {A}_M(p)$. Consequently, the conjunctive condition $q,r\in \mathcal {A}_M(p)$ eliminates not only those mixed breakpoints that are not dominant, but also those calculated on potentially incorrect *P*(*ij*, *q*) from Eq. (25). Note that, unlike the inputs-only case of Sect. 3.1, two breakpoints between any $\{ij,q\}$ and $\{ij,r\}$ can occur, because $f_{\{ij,q\}}(p)$ and $f_{\{ij,r\}}(p)$ are convex or concave functions (see Proposition 3.3.7) which can intersect at two points. The endpoints of $\hat{{\varPhi }}$ also represent mixed dominance breakpoints, since for $p\in \{b_l,b_u\}$, given $\mathcal {A}_M(p)=\{ij,q\}$, either *ij*, *q* or *P*(*ij*, *q*) change at *p*, creating a breakpoint. $\square$

#### Proposition 3.3.6

For a *p*-interval $${\varPhi }=[b_l,b_u]$$, $$(\forall p\in {\varPhi })\ \mathcal {A}_I(p)=ij$$:

- $(\forall p \in {\varPhi }_I\subseteq I_{LU})$, *ij* feasible and $\mathcal {A}^*(p)\in \{\mathcal {A}_I(p),\mathcal {A}_D\}$ with dominance breakpoint
  28 $$\begin{aligned} \left\{ p\in {\varPhi }_I\bigg |\ ij\asymp _p \mathcal {A}_D:=\alpha \Leftrightarrow \gamma _{ij}(p)=\gamma _\alpha \Leftrightarrow p= \frac{C_i(\gamma _q-\gamma _j)-C_j(\gamma _q-\gamma _i)}{\gamma _i-\gamma _j} \right\} \subseteq \mathcal {B}. \end{aligned}$$
- $(\forall p\in \hat{{\varPhi }})$, for $\hat{{\varPhi }}\in \{{\varPhi }_M;\ {\varPhi }_I \not \subseteq I_{LU}\}\subseteq {\varPhi }$, $\mathcal {A}^*(p)\in \{\mathcal {A}_D,\mathcal {A}_M(p)\}$ with dominance breakpoints 29

#### Proof

(a) By construction, $(\forall p\in {\varPhi }_I\subseteq I_{LU})\ \mathcal {A}_I(p)\succeq _p \mathcal {A}_M(p)$ and thus $\mathcal {A}^*(p)\in \{ij,\mathcal {A}_D\}$. The breakpoint in Eq. (28) follows directly from Lemma 3.3.3.

(b) The breakpoint condition in Eq. (29) is similar to the one of Lemma 3.3.3, but with the input active set replaced by $\mathcal {A}_D = \alpha$. The associated dominance breakpoint $b_{\{ij,q\},\alpha }$ is also found analogously as in Lemma 3.3.3 (details omitted for brevity). The use of the *p*-independent expression for *P*(*ij*, *q*) in Eq. (25) for every $q\in {\varTheta }({\varPhi })$ is justified by the same arguments as in the proof for Proposition 3.3.5.ii (mixed set only on one side of any potential breakpoint). $\square$

#### Proposition 3.3.7

*(Derivatives of objective function w.r.t.* *p* *and convex/monotone properties)* For fixed *p* and mixed active triple $A=\{i,j,q\}=\{ij,q\},\ i,j\in T_X,\ q\in T_Z$ in Problem P–3.4,

30 $$\begin{aligned} {\frac{\partial ^{n} f_A}{\partial p^{n}}}=(-1)^nn! \frac{D\big (P(ij,q)-C_q\big )}{(C_i-C_j)(p-C_q)^{n+1}}\,\cdot \, \bigg (C_q(\gamma _j-\gamma _i) + C_j(\gamma _i-\gamma _q)+ C_i(\gamma _q-\gamma _j)\bigg ),\ \end{aligned}$$

which implies $f_A(p)$ is monotone convex/concave:

- If $p>C_q$ then $\mathop {\mathrm {sgn}}({\frac{\partial f_A}{\partial p}})\not = \mathop {\mathrm {sgn}}({\frac{\partial ^{2} f_A}{\partial p^{2}}})$ and $f_A(p)$ is concave increasing or convex decreasing.
- If $p<C_q$ then $\mathop {\mathrm {sgn}}({\frac{\partial f_A}{\partial p}}) = \mathop {\mathrm {sgn}}({\frac{\partial ^{2} f_A}{\partial p^{2}}})$ and $f_A(p)$ is concave decreasing or convex increasing.

#### Proof

Proof in “Appendix A”. $\square$

#### Theorem 3.3.8

(*p*-Parametric structure of the optimal objective function $f^*(p)$*)*

Consider a given *p*-interval ${\varPhi }$ between two consecutive dominance breakpoints for Problem P–3.4. Functions $f^*,\ \mathcal {A}^*,\ \mathcal {A}_I,\ \mathcal {A}_M$ are *p*-parametric with the following cases,

- (direct) $(\forall p\in {\varPhi })\ \mathcal {A}^*(p)=\mathcal {A}_D=\{l,r\} \text { or } r\ \Rightarrow f^*|_{{\varPhi }}(p)$ *is constant*,
- (input) $(\forall p\in {\varPhi })\ \mathcal {A}^*(p)=\mathcal {A}_I(p)=ij\ \qquad \ \ \Rightarrow f^*|_{{\varPhi }}(p)$ *is linear*,
- (mixed) $(\forall p\in {\varPhi })\ \mathcal {A}^*(p)=\mathcal {A}_M(p)=\{ij,q\}\ \Rightarrow f^*|_{{\varPhi }}(p)$ *is monotone convex/concave*,

where $(\forall p\in {\varPhi })$ $i,j\in T_X,\ l,r,q\in T_Z$ are fixed.

#### Proof

Case (a) follows from Theorem 3.2.7 and the independence of the results therein w.r.t. *p*; Case (b) from Lemma 3.1.2 and Proposition 3.1.5; Case 3 from Theorem 3.3.1 and Proposition 3.3.7. $\square$

#### Theorem 3.3.9

(Optimal solution and dominance breakpoints for I${+}\mathrm{H}{-}1{-}1$ subclass)

For Problem P–3.4, let $\mathcal {B}_{IM}\in B_I\cup B_M$ and exclude from $\mathcal {B}_{IM}$ any redundant breakpoints with the same active sets on both sides. Then

31 $$\begin{aligned} f^*=\max \left( \max \limits _{p\in \mathcal {B}_{IM}} f^*(p),f^*_{\mathcal {A}_D}\right) , \end{aligned}$$

with the optimal objective value $f^*_{\mathcal {A}_D}$ for the dominant direct active set acting as a threshold. Both $f^*$ and a full description of $f^*(p)$ via all dominance breakpoints $\mathcal {B}$ ($\mathcal {B}_{IM}$ plus all dominance breakpoints with $\mathcal {A}_D$) can be obtained in strongly-polynomial time $O(I^3+ H^3)$.

#### Proof

Denoting by $T(\cdot )$ the time-complexity of calculating result $(\cdot )$,

32 $$\begin{aligned} \begin{aligned} T(f^*)&= T(\mathcal {B}_{I}) + T(\mathcal {B}_{M}) + T(\mathcal {A}_D) = T ({{\textit{Eq.}} \, (11\mathrm{a})}) + T ({{\textit{Eq.}} \, (27)}) + T ({{\textit{Eq.}} \, (15)})\\&= O(I^3) + O(H^3) + O(H^2) = O(I^3+H^3). \end{aligned} \end{aligned}$$

Eq. (27) can be solved in $O(H^3)$ because all mixed dominance breakpoints for all *p*-intervals ${\varPhi }$ can be found in one pass of complexity $O(H^3)$ and then assigned to each *p*-interval. Since $T(Eq. \, (29))+ T(Eq. \, (28))=O(H^2)$, $T(\mathcal {B}) = T(f^*)$. $\square$

**Fig. 6:**
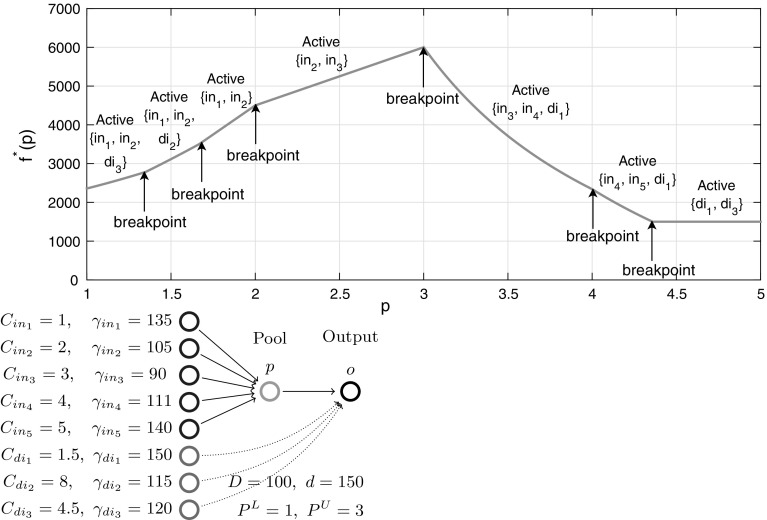
Optimal objective function $f^*(p)$ versus pool concentration *p* for a one pool, one output network with five inputs and three directs (parametrized with concentrations/costs). The objective is a piecewise-monotone convex/concave/linear function of the pool concentration

Figure 6 illustrates the I${+}\mathrm{H}{-}1{-}1$ subclass with a numerical example showcasing the implications of Theorem 3.3.8. For a chosen parametrization of five inputs (same inputs as in Fig. 4) and three directs with quality constraints, the *p*-parametric function $f^*(p)$ reveals additional breakpoints compared to Fig. 4 between mixed and input active sets and between mixed and direct active sets. The structure is still piecewise-monotone, but is extended to piecewise *p*-intervals exhibiting convexity or concavity, e.g. when $3\le p\le 4$ where mixed active set $\{in_3,in_4,di_1\}$ is dominant.

Similar to the results in Sect. 3.1, the coupling between the piecewise structure and sparse active sets (up to a mixed node triple) is still present, allowing a full description of the *p*-parametric optimal solution space. Section 4 explicitly uses this full description to find optimal solutions in strongly-polynomial time for a multiple outputs instance.

#### Remark 3.3.10

*(From analytical solutions/sparsity/piecewise structure to non-sparse LP)*

Section 3 finds analytically the optimal solution for a I+H-1-1 pooling topology with Assumption 2.2. Beyond the knowledge of sparsity in the Problem P–3.4 LP, this section identifies the active feeds in the *p*-parametric optimal objective $f^*(p)$, and as a result the piecewise-monotone $f^*(p)$ structure. In fact, these results are tightly conditioned by Assumption 2.2, as follows:

- *Reinstating constraints on feed availability (or analogously pool capacity)* will erode solution sparsity proportionately to tightness of the flow bounds on dominant feeds. For fixed *p*, if the dominant active set reaches its upper flow bounds, the next-in-line dominant active set would send flow and so on, recursively. If the bounds on cheaper feeds are very tight, this effect would create a hierarchy of dominant active sets participating in the solution. Therefore, the tighter the flow bounds, the more active feeds, and the less sparsity. The piecewise function $f^*(p)$ would hence have more breakpoints accounting for dominance relations and optimal balancing between all active sets in the dominance hierarchy, not just the top active set. Moreover, because $f^*(p)$ would represent an addition over a hierarchy of active sets, $f^*(p)$ can be piecewise non-monotone as in Sect. 4. Equally importantly, balancing the flows among the hierarchy of dominant active sets under flow constraints is an inherent LP, i.e. not solvable analytically.
- *Introducing multiple qualities (*$K>1$*)* keeps the problem as an LP, but its polynomial complexity increases in line with *K* as the dominant active set cardinality becomes $K{+}1$ (this extension is possible for one pool, one output topologies).
- *Relaxing the fixed product demand assumption* implies the same solution with product demand reaching its upper limit if the problem is (assumed) feasible.

## Subclass I$\,+\,\mathrm{H}{-}1{-}$J: one pool, multiple outputs

This section extends the analysis in Sect. 3 with Assumption 2.2 to $I{+}H$ feeds (*I* inputs, *H* directs), one pool and multiple *J* outputs. Again, for simplicity of notation, single index *l* is dropped via the notation transformations $T_X \leftarrow \{i:(i,l)\in T_X\}$, $T_Y \leftarrow \{j:(l,j)\in T_Y\}$. This leads to Problem P–4.10, where for each output only connected to directs surplus variables $y_j\ \forall j\in \overline{1,J}\setminus T_Y$ are introduced and set to 0 as a surplus condition. Note that eliminating *p* and $y_j\ \forall j\in T_Y$ from Problem P–4.10 does not produce a linear program as in Sect. 3, but instead a bilinear problem that can be non-convex.

To analyze Problem P–4.10, Theorem 4.1 first proves its equivalence to Problem P–4.11. The result allows additively decomposing Problem P–4.11 over outputs into sub-problems P–4.11-*j*, which are all *p*-parametric and equivalent to the subclass I+H-1-1 studied in Sect. 3. Definition 4.2 then extends dominance breakpoints and dominant active sets for a multiple outputs problem setting. Proposition 4.3 shows that the composed master Problem P–4.11 can present *p*-parametric non-monotonicity or non-convexity on specific *p*-intervals. This hurdle is cleared by Theorem 4.4, which finds in polynomial time all stationary points on non-monotone breakpoint intervals by solving a univariate rational polynomial. Finally, Corollary 4.5 offers a strongly-polynomial worst-case time complexity for solving Problem P–4.11 to optimality.

The section concludes by Remark 4.6, showing the I+H-1-J subclass lies on the *P / N P* boundary due to the fact that any relaxation of the assumptions made leads to an NP-hard problem. 
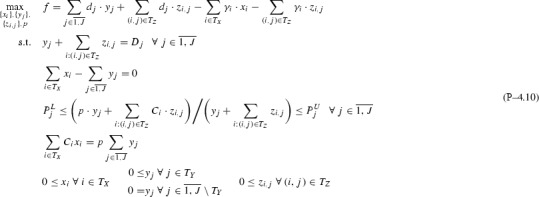

**Fig. 7:**
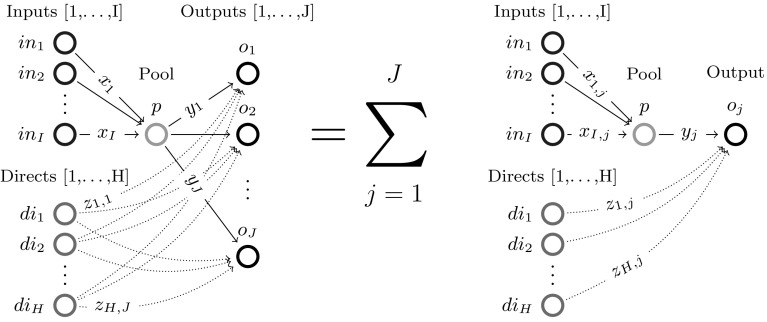
*p*-Parametric additive decomposition of a multiple outputs problem into a sum of one output problems based on which output feed flows arrive at

### Theorem 4.1

(*p*-Parametric additive decomposition over multiple outputs)

As visualized in Fig. 7, Problem P–4.10 can be reformulated as a Problem P–4.11 of maximizing the total sum of *J* *p*-parametric optimal objectives over *p*, each associated to a one output sub-problem P–4.11-*j* (same type as Problem P–3.4).
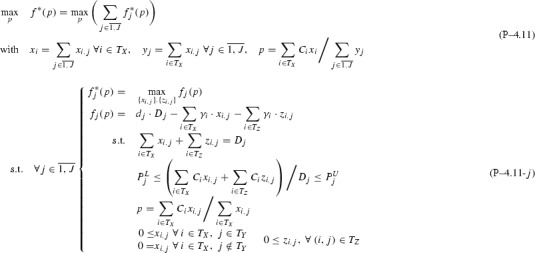


### Proof

Each $x_i\ \forall i\in T_X$ can be split into a sum of flows $x_{i,j}\ \forall j\in \overline{1,J}$, with each $x_{i,j}$ representing the flow output *j* gets via the pool from input *i*. This allows similar output-based splits for the two constraints in Problem P–4.10 that apply jointly over all outputs, i.e. flow and quality balance:

$$\begin{aligned} x_i =\sum \limits _{j\in \overline{1,J}}x_{i,j} \quad \forall i\in T_X\quad \Rightarrow \quad \sum _{i\in T_X}x_{i,j} - y_j = 0\quad \forall j\in \overline{1,J}, \sum _{i\in T_X}C_ix_{i,j} = p\cdot y_j\quad \forall j\in \overline{1,J}. \end{aligned}$$

Furthermore, the objective *f* as a function of *p* in Problem P–4.10 can be rewritten as,

$$\begin{aligned} f(p) = \sum _{j\in \overline{1,J}} \bigg ( d_j\cdot y_j+\sum _{i:(i,j)\in T_Z}d_j\cdot z_{i,j}-\sum _{i\in T_X}\gamma _i\cdot x_{i,j}-\sum _{i:(i,j)\in T_Z}\gamma _i\cdot z_{i,j}\bigg ):= \sum _{j\in \overline{1,J}}f_j(p), \end{aligned}$$

where each $f_j$ contains a different set of variables and parameters for a fixed *j* except for common variable *p* which it is parametrized on. In the context of maximizing *f*(*p*), we have:

$$\begin{aligned} \begin{aligned} \max _{\{x_i\},\{y_j\},\{z_{i,j}\},p} f&= \max _p\bigg (\max _{\{x_i\},\{y_j\},\{z_{i,j}\}} f(p)\bigg ) = \max _p\bigg (\max _{\{x_{i,j}\},\{y_j\},\{z_{i,j}\}} f(p)\bigg )\\&=\max _{p} \bigg (\max _{\{x_{i,j}\},\{y_j\},\{z_{i,j}\}} \sum _{j\in \overline{1,J}} f_j(p) \bigg ) = \max _{p}\bigg ( \sum _{j\in \overline{1,J}} \max _{\{x_{i,j}\},\{z_{i,j}\}} f_j(p) \bigg ) \\&= \max \limits _{p}\bigg ( \sum \limits _{j\in \overline{1,J}} f_j^*(p)\bigg ). \end{aligned} \end{aligned}$$

Finally, separating out both the objective and constraints parts for all $j\in \overline{1,J}$ from Problem P–4.10, we obtain a collection of *J* sub-problems P–4.11-*j*, all parametric on and therefore linked via a common *p* but otherwise independent. However, as shown in Sect. 3.3, each *p*-parametric sub-problem P–4.11-*j* can be solved analytically using the piecewise structure of $f_j^*(p)$, and each solution can be used directly towards solving the master Problem P–4.10 linking all *J* sub-problems. Note that, for each sub-problem P–4.11-*j* $\forall j\in \overline{1,J}$, $y_j$ is eliminated as in Problem P–3.4, with the surplus condition enforced through the *x* variables set to 0; when $j\notin T_Y$, the sub-problem is thus a directs-only one. $\square$

### Definition 4.2

*(Dominance breakpoints and dominant active sets)*

- Let $\mathcal {B}_J=\bigcup _{j\in \overline{1,J}}\mathcal {B}_j$ be the joint dominance breakpoint set for Problem P–4.11-*j* over all *J* outputs/sub-problems, with $\mathcal {B}_j$ the set of all dominance breakpoints for the *j*-th sub-problem P–4.11-*j*, found as in Sect. 3.
- Let ${\varPhi }_J$ denote any closed interval with two consecutive elements in $\mathcal {B}_J$ as endpoints.
- Let $\mathcal {A}^*_j(p)$ denote the dominant active set at *p* for the *j*-th sub-problem P–4.11-*j*, as found in Theorem 3.3.8; by construction, $\mathcal {A}^*_j(p)$ remains constant over ${\varPhi }_J$ s.t. $\forall p\in {\varPhi }_J\ \mathcal {A}^*_j(p)=\mathcal {A}^*_j({\varPhi }_J)$.

### Proposition 4.3

In Problem P–4.11, for $\forall p\in {\varPhi }_J$, $f^*(p)$ can be non-monotone or non-convex.

### Proof

From Theorem 3.3.8, for a given ${\varPhi }_J$, $(\forall j\in \overline{1,J})(\forall p\in {\varPhi }_J)\ f_j^*(p)$ can be either constant, linear or monotone convex/concave depending on dominant active set $\mathcal {A}^*_j({\varPhi }_J)$. Consider a two-output Problem P–4.11 with,

$$\begin{aligned} f^*(p)= f_1^*(p)=f_{j_1}^*(p)+f_{j_2}^*(p), \end{aligned}$$

and $f_{j_1}^*(p)$ concave increasing and $f_{j_2}^*(p)$ concave decreasing. Then

$$\begin{aligned} \exists p_1\in {{\mathrm{int}}}({\varPhi }_J) \text { s.t. }\frac{\partial f^*(p)}{\partial p} = \frac{\partial f^*_{j_1}(p)}{\partial p} + \frac{\partial f^*_{j_2}(p)}{\partial p}=0, \end{aligned}$$

meaning $f^*(p)$ has a local maximum at $p_1$ and is non-monotone. Now consider a four-output Problem P–4.11 with $f^*(p)=f_1^*(p)+f_2^*(p)$ with $f_2^*(p)$ constructed analogously as $f_1^*(p)$ but having a local maximum at $p_2\in {\varPhi }_J,\ p_2\not =p_1$. In this case, $f^*(p)$ is multi-modal with at least two local maxima and therefore non-convex. $\square$

### Theorem 4.4

Assuming Problem P–4.11 has all parameters rational, given interval ${\varPhi }_J$, then finding $f^*|_{{\varPhi }_J}=\max _{p\in {\varPhi }_J}(f^*(p))$ requires finding all stationary points of $f^*(p)$ over ${\varPhi }_J$ by solving a univariate rational polynomial of maximum degree $2\cdot |T_Y|$, i.e.:

34 $$\begin{aligned} \begin{aligned} \frac{\partial f^*(p)}{\partial p}&= \bigg (\sum \limits _{\begin{array}{c} j\in T_Y,\\ \text {s.t. }\mathcal {A}^*_j=\{i,k,q\}\\ i,k\in T_X,\ (q,j) \in T_Z \end{array}} \frac{D_j\big (C_q- P^j(ik,q)\big )}{(C_i-C_k)(p-C_q)^2}\cdot \big (C_q(\gamma _k-\gamma _i) + C_k(\gamma _i-\gamma _q)+ C_i(\gamma _q-\gamma _k)\big )\bigg ) \\&\quad +C=0, \end{aligned} \end{aligned}$$

where *C* is a constant and $P^j(\cdot ,\cdot )$ is defined as in Eq. (25) but for bounds $P_j^L,\ P_j^U$. The polynomial in Eq. (34) can be solved in strongly-polynomial time with respect to $T_Y,\ |T_Y|\le J$.

### Proof

Section 3 implies that for Problem P–4.11,

35 $$\begin{aligned} \begin{aligned} \frac{\partial f^*(p)}{\partial p}=&\sum _{j\in \overline{1,J}} \frac{\partial f_j^*(p)}{\partial p} \overset{{\text {Section 3.2}}}{=} \sum _{j\in T_Y} \frac{\partial f_j^*(p)}{\partial p} \\ =&\sum \limits _{\begin{array}{c} j\in T_Y,\\ \text {s.t. }\mathcal {A}^*_j=\{i,k,q\}\\ i,k\in T_X,\ (q,j) \in T_Z \end{array}} \frac{\partial f_j^*(p)}{\partial p} + \sum \limits _{\begin{array}{c} j\in T_Y,\\ \text {s.t. }\mathcal {A}^*_j=\{i,k\}\\ i,k\in T_X \end{array}} \frac{\partial f_j^*(p)}{\partial p} \overset{\begin{array}{c} {\text {Theorem 3.3.8}},\\ {\text {Lemma 3.1.2}} \end{array}}{=} {{\textit{Eq.}}\, (34)}. \end{aligned} \end{aligned}$$

The value $f^*|_{{\varPhi }_J}$ corresponds to the maximum objective function value evaluated at all stationary points. To find all roots/stationary points of $f^*(p)\ \forall p\in {\varPhi }_J$, the common denominator can be multiplied out in Eq. (34) to form a polynomial. The resulting polynomial from Eq. (34) will have maximum degree $2\cdot T_Y$ if $\forall j\in T_Y\ \mathcal {A}^*_j({\varPhi }_J)=\{i,k,q\},\ i,k\in T_X,\ (q,j) \in T_Z$ and if $q_1\not =q_2$ given $q_1\in \mathcal {A}^*_{j_1}({\varPhi }_J),\ q_2\in \mathcal {A}^*_{j_2}({\varPhi }_J),\ (q_1,j),(q_2,j)\in T_Z$. Furthermore, by assumption, the polynomial in Eq. (34) has rational coefficients. Consequently,

36 $$\begin{aligned} \frac{\partial f^*(p)}{\partial p} = \sum \limits _{0\le i\le 2\hat{J},\ \hat{J}\le T_Y} a_i\cdot p^i,\qquad \forall i\ a_i\in \mathbb {Q}, \end{aligned}$$

which, as a rational univariate polynomial, can be solved deterministically in strongly-polynomial time, using for example the algorithm in [38] with worst-case time bound $T(\text {UnivPoly})$,

37 $$\begin{aligned} \begin{aligned} T(\text {UnivPoly})&= O\left( (2|T_Y|)^{12} + (2|T_Y|)^9 \left( \log \left| \frac{\partial f^*(p)}{\partial p} \right| \right) ^3\right) , \\ \text {where }\left| \frac{\partial f^*(p)}{\partial p} \right|&=\left( \sum \limits _i a_i^2 \right) ^{1/2}. \end{aligned} \end{aligned}$$

$$\square $$

### Corollary 4.5

*(Strongly-polynomial time complexity)*

Problem P–4.11 $\Leftrightarrow$ Problem P–4.10 with all parameters rational is solved in strongly-polynomial worst-case time

38 $$\begin{aligned} O((I^3+H^3)\cdot J) + O(I\cdot H^2)\cdot \left( O(\text {UnivPoly}) + O(J)\right) , \end{aligned}$$

where $O(\text {UnivPoly})$ is polynomial w.r.t. $|T_Y|<=J$ (see Theorem 4.4, Eq. (37)).

### Proof

In the worst case, on all intervals between two joint dominance breakpoints in $\mathcal {B}_J$ the additive decomposition over outputs in Problem P–4.11 requires solving a univariate rational polynomial as in Theorem 4.4. Denoting by $T(\cdot )$ the time-complexity of calculating result $(\cdot )$, this implies

39 $$\begin{aligned} \begin{aligned} T(f^*)&= T(\mathcal {B}_J) + O(|\mathcal {B}_J|)\cdot \left( O(\text {UnivPoly}) + O(J)\right) \\&= O(I^3+H^3)\cdot O(J) + O(|\mathcal {B}_J|)\cdot O(\text {UnivPoly}). \end{aligned} \end{aligned}$$

Eq. (39) accounts for both the size of $\mathcal {B}_J$ and the time to find $\mathcal {B}_J$ assuming for simplicity $|T_Y|=J$. Eq. (39) also includes objective evaluations at maximum *J* stationary points found after applying Theorem 4.4 (time $O(\text {UnivPoly})$) for any breakpoint interval. Furthermore, due to Theorem 3.3.9, $\max |\mathcal {B}_J|\Rightarrow \max |\mathcal {B}_{IM}|$ when all breakpoints between input and mixed sets are dominant. Assuming a breakpoint between the dominant mixed/input set and the dominant direct set on any interval between two consecutive elements of $\mathcal {B}_{IM}$ does not change the order of $|\mathcal {B}_{IM}|$. Therefore,

40 $$\begin{aligned} O(|\mathcal {B}_J|) = O(|\mathcal {B}_{IM}|) = O(|\mathcal {B}_{I}|)\cdot O(|\mathcal {B}_{M}|) = O(I\cdot H^2), \end{aligned}$$

since on each interval between two input dominance breakpoints there can be mixed dominance breakpoints between a pair of mixed sets, therefore between a pair of directs (see Proposition 3.3.5). Finally, combining Eqs. (39) and (40) results in Eq. (38). $\square$

**Fig. 8:**
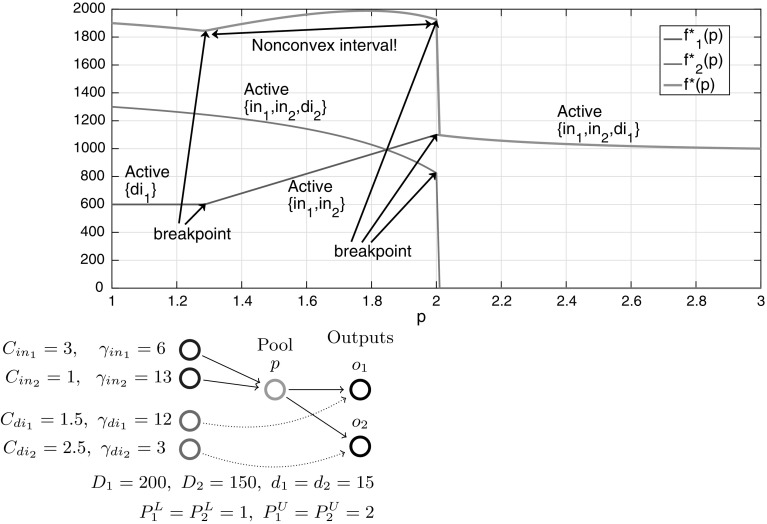
Optimal objective function $f^*(p)$ versus pool concentration *p* for a one pool, two-output network with two inputs and two directs (parametrized with concentrations/costs). The objective $f^*(p)=f^*_1(p) + f^*_2(p)$ is a sum of the objectives corresponding to one output sub-problems, and is a piecewise (possibly non-monotone/non-convex) function of the pool concentration

Fig. 8 illustrates the I+H-1-J subclass analysis with a numerical example showcasing the implications of Proposition 4.3 and Theorem 4.4. For a chosen parametrization of two inputs, directs and outputs with quality constraints for each output in Fig. 8, the *p*-parametric function $f^*(p)$ is decomposed additively in the optimal objectives of the one output sub-problems. Hence, in the particular example, $f^*(p)$ has a non-monotone piecewise section as proven in Proposition 4.3 for $p\in (1.3,2)$ with a stationary point which can be deterministically found via Theorem 4.4.

### Remark 4.6

(A new *P* / *NP* *boundary point for standard pooling)*

The Sect. 4 results address a gap in the literature for the *P* / *NP* boundary for standard pooling problems. The time-complexity gap [4, Corollary 2] occurs between the single quality I+H-L-J class that is strongly NP-hard and the I-1-J class with a bounded number of qualities that is polynomial-time solvable. Theorem 4.4 and Corollary 4.5 show that the single quality I+H-1-J class restricted by Assumption 2.2 is strongly-polynomial. Any relaxation of Assumption 2.2 is NP-hard:

- *Reinstating constraints on feed availability/pool capacity* for any particular $j\in T_Y$ implies the mixed active set term in Eq. (34) gets split into a hierarchy of potential mixed active sets (see Remark 3.3.10) each with total flow an unknown proportion of $D_j$. This hierarchy of sets leads to a bivariate rational polynomial which is NP-hard to solve [25].
- *Introducing multiple qualities (*$K>1$*)* implies variables $p_k,\ k\in \overline{1,K},$ are not independent - a specific pool concentration in one quality restricts the concentration range in another. Consequently, Eq. (34) becomes an NP-hard multivariate polynomial system.
- *Relaxing the fixed product demand assumption* - if any of $D_j\ \forall j\in \ T_Y$ are not fixed but unknown, then Eq. (34) becomes an NP-hard bivariate polynomial system.
- *Extending to the full topology* $I{+}H{-}L{-}J$, again Eq. (34) translates to a coupled multivariate polynomial system when two pools send non-zero flow to the same output (as in Sect. 5, Theorem 5.2) and one of the two pools also sends non-zero flow to a different output.

In summary, when feed to output connections (directs) are considered, the I$+\mathrm{H}{-}1{-}\mathrm{J}$ class following Assumption 2.2 lies on the *P* / *NP* boundary, and can be solved analytically as shown in this section despite $f^*(p)$ being piecewise non-monotone or non-convex.

### Remark 4.7

*(Haverly* [33] *is strongly-polynomially solvable)* The Haverly [33] instances, i.e. the first set of three pooling problems in the literature, are part of the single-quality I+H-1-J class following Assumption 2.2. We can obtain their exact solutions analytically in strongly-polynomial time!

### Remark 4.8

*(Connection to queueing theory)* Woodside and Tripathi [60] report similar, piecewise-monotone structure in a central processor queueing problem where workstation files are assigned to file servers. The Woodside and Tripathi [60] proofs cannot be directly applied to standard pooling, but the similarity recalls the deep connection between pooling and queueing.

## Subclass I$+\mathrm{H}{-}\mathrm{L}{-}1$: multiple pools, one output

This section extends the analysis in Sect. 3 with Assumption 2.2 to $I{+}H$ feeds (*I* inputs, *H* directs), *L* pools and one output. Again, for simplicity of notation, single index *j* is dropped via the notation transformations $T_Z \leftarrow \{i:(i,j)\in T_Z\}$, $T_Y \leftarrow \{l:(l,j)\in T_Y\}$. This leads to Problem P–5.12, where eliminating variables $p_l,y_l\ \forall l\in T_Y$ results in an LP as for Problem P–3.4 in Sect. 3, limited to a maximum cardinality of four in terms of the $x,\ z$ variables. We further identify this solution analytically, understanding pool sparsity and the parametric structure of the optimal objective in the process. To analyze Problem P–5.12, Definition 5.1 first introduces active pools. Theorem 5.2 then finds a maximum of two active pools contribute to the optimal solution and further shows all cases induced are parametric on pools concentrations. Furthermore, Theorem 5.3 proves all cases involved in Theorem 5.2 in fact reduce to the I+H-1-1 subclass studied in Sect. 3. Finally, Corollary 5.4 offers a strongly-polynomial bound on solving Problem P–5.12 and the section concludes with an illustrative numerical example.

### Definition 5.1

*(Active pools)*

- Let the $L=\vert T_Y \vert$ pools in Problem P–5.12 be denoted by *i* with concentration $p_i$, $\forall i\in \overline{1,L}$.
- An active pool has incoming and outgoing flows strictly non-zero. A non-active pool $l\in T_Y$ has well-defined concentration by assuming only $y_l=0$ but $x_{i,l}\not =0,\ \forall i:(i,l)\in T_X$. However, any non-active pool is disconnected via $y_l=0$ from the output, does not influence objective function *f*, and can be removed along with any of its flow connections from Problem P–5.12.

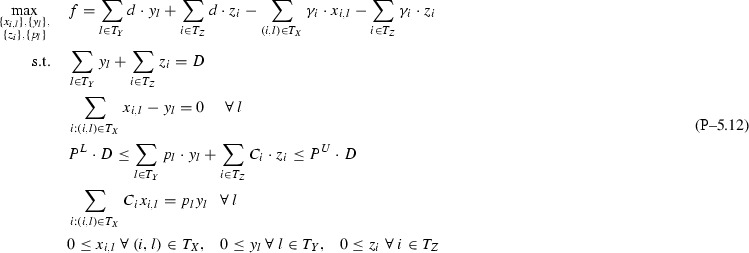


### Theorem 5.2

*(Pool sparsity and pool-parametric objective function)*

For fixed $p_l\ \forall l\in T_Y$, at optimality, Problem P–5.12 has a maximum of two active pools with the optimal objective function parametric on their concentrations:

41 $$\begin{aligned} \max _{\begin{array}{c} \{x_{i,l}\}, \{y_{l}\},\\ \{z_{i}\}, \{p_l\} \end{array}} f = {\left\{ \begin{array}{ll}\begin{aligned} \max \limits _{\begin{array}{c} \{x_{i,m}\},\{x_{i,n}\} \end{array}} &{} f(p_m,p_n),&{} &{}\text {if }m,n\in T_Y \text { active, } \quad \ \ T_Y\setminus \{m,n\} \text { not active,}\\ \max \limits _{\begin{array}{c} \{x_{i,m}\}, \{z_{i}\} \end{array}}&f(p_m),&\,&\text {if } m\in T_Y \text { active or not, } T_Y\setminus \{m\} \text { not active.} \end{aligned}\end{array}\right. } \end{aligned}$$

### Proof

Variables $p_l\ \forall l\in T_Y$ and $y_l\ \forall l\in T_Y$ can be substituted out from Problem P–5.12 using the penultimate (quality balances) and second (flow balances) constraint types, respectively. Hence, Problem P–5.12 becomes parametric on $p_l\ \forall l\in T_Y$ with optimal objective

42 $$\begin{aligned} \max _{\begin{array}{c} \{x_{i,l}\}, \{y_{l}\}, \{z_{i}\}, \{p_l\} \end{array}} f =\max _{\begin{array}{c} \{x_{i,l}\}, \{z_{i}\} \end{array}} f(p_1,\dots ,p_L). \end{aligned}$$

For fixed pool concentrations $p_l\ \forall l\in T_Y$, all pools have fixed optimal cost $\gamma _l$ (obtained as $\gamma _l=\gamma _{ij}(p_l)$ with $\mathcal {A}_I(p_l)=ij$ for the I-1-1 subclass according to Sect. 3.1) and thus behave like additional directs sending flow directly to the output. Consequently, Theorem 3.2.7 for the directs-only subclass from Sect. 3.2 applies, implying that maximum two nodes among pools and directs are active.

**Fig. 9:**
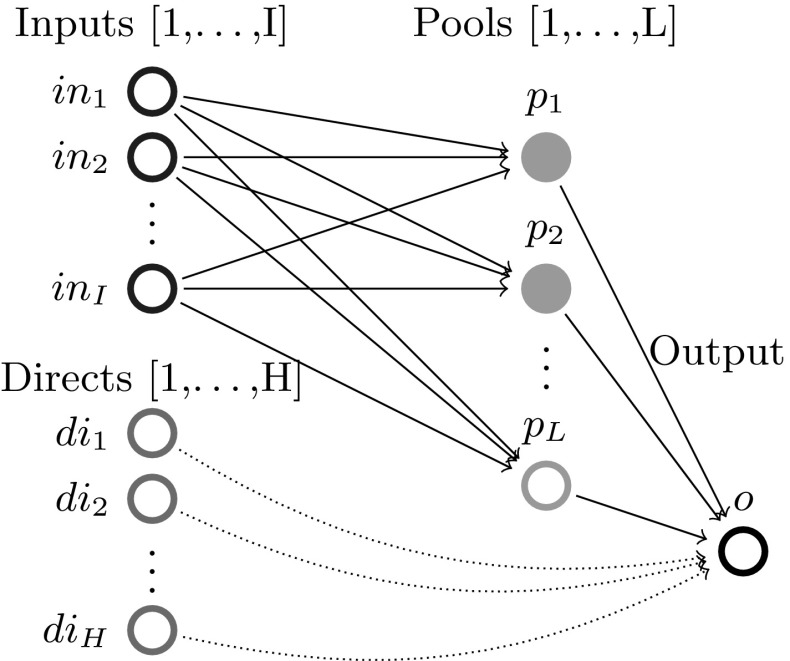
Two active pools (filled nodes) in an I+H-L-1 instance

The case with two pools active (see Fig. 9) is treated separately in Eq. (41) as a two pool parametric restriction of Eq. (42) where no directs are active (therefore associated flow variables $\{z_i\}$ can be eliminated). The second case in Eq. (41) aggregates the cases with maximum one pool active, and corresponds directly to the class of Problems P–3.4 solved in Sect. 3.3. $\square$

### Theorem 5.3

*(Solution for two active pools at an input dominance breakpoint)*

The solution of Problem P–5.12 with two active pools *m*, *n* is found at $(p^b_m,p^b_n)$, where $(\forall i\in \{m,n\})\ p^b_i$ is an input dominance breakpoint along $p_i$ in a single *i* pool I-1-1 subclass (Sect. 3.1), i.e.

43 $$\begin{aligned} \begin{aligned} \max \limits _{\begin{array}{c} \{x_{i,m}\},\{x_{i,n}\} \end{array}}&f(p_m,p_n) = \max \limits _{(p^b_m,\ p^b_n)\in \mathcal {B}_{I(m)} \times \mathcal {B}_{I(n)}} f^*(p^b_m, p^b_n), \\ \text {where }&\mathcal {B}_{I(r)} {\text { as in Theorem 3.1.6, Eq. (11a) for }} f^*= \max \limits _{p_r} f^*(p_r) = \max \limits _{\begin{array}{c} \{x_{i,r}\}, \{z_{i}\} \end{array}} f(p_r).\\ \end{aligned} \end{aligned}$$

### Proof

Suppose concentration $p_n$ is fixed which implies pool *n* acts as an additional direct with fixed concentration and cost (optimal). Since $p_m, p_n$ are independent, parametric optimal objective $f^*(p_m,p_n)$ behaves like $f^*(p_m)$ in a single *m* pool problem where *n* acts as a direct, not as a pool. Pools *m*, *n* both active implies that $\forall p_m$ the dominant active set for $f^*(p_m)$ is a mixed active set $\{i,j,n\}$ with *n* as a direct and $\{i,j\}$ the dominant active input set sending flow to *m*. Since *n* must be active and therefore part of the dominant active set, a change in the dominant active set can only occur with a change of dominant active input set $\{i,j\}$, independently of *n* and therefore fixed value $p_n$. Consequently, the dominance breakpoints of $f^*(p_m,p_n)$ w.r.t. $p_m$ occur independently of the value of $p_n$ and are always breakpoints between dominant active input sets for pool *m*. The vice-versa independence also holds. Excluding input dominance breakpoint pairs of $f^*(p_m,p_n)$ w.r.t both $p_m,p_n$ in $\mathcal {B}_{I(m)} \times \mathcal {B}_{I(n)}$, function $f^*(p_m,p_n)$ is linear (Lemma 3.1.2) in at least one parameter (with gradient non-zero). Hence, Eq. (43) follows. $\square$

**Fig. 10:**
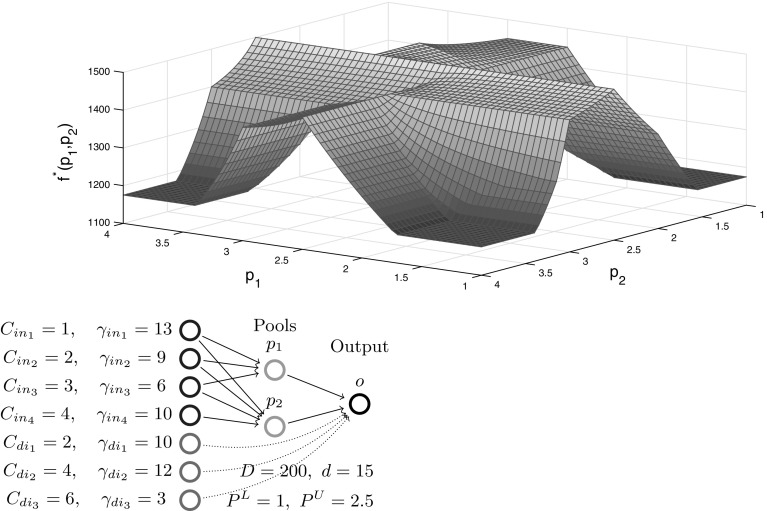
Parametric optimal objective function $f^*(p_1,p_2)$ versus pools concentrations $p_1,p_2$ for a two-pool, one output network with four inputs and three directs (parametrized with concentrations/costs). The objective is a piecewise-monotone convex/concave/linear function w.r.t. $p_1$ or $p_2$ individually

### Corollary 5.4

*(Strongly-polynomial time complexity)*

Problem P–5.12 is solved in strongly-polynomial worst-case time

44 $$\begin{aligned} O\left( (I^3+ H^3)\cdot L + I^2\cdot L^2\right) \end{aligned}$$

### Proof

According to Theorems 5.3 and 3.1.6, the first case in Eq. (41) for two given active pools involves $2\cdot O(I^3)$ time for finding the two sets of input dominance breakpoints and $O(I^2)$ for evaluating the optimal parametric objective function at all breakpoint pairs. Since there are $$\left( {\begin{array}{c}L\\ 2\end{array}}\right) $$ possible pairs of active pools, the total time for the first case of Eq. (41) is $O(I^3\cdot L) + O(I^2\cdot L^2)$.

The second case in Eq. (41) for one possibly active pool is equivalent to solving Problem P–3.4 (Theorem 3.3.9) and there are *L* active pool choices for a total time $O((I^3{+} H^3)\cdot L)$.

Adding the two cases of Eq. (41) yields the time complexity in Eq. (44). $\square$

Figure 10 illustrates the I+H-L-1 subclass with a numerical example showcasing the implications of Theorem 5.2 and Theorem 5.3. For a chosen parametrization as in Fig. 10, the parametric function $f^*(p_1,p_2)$ reveals portions of the domain where only one pool is active (around the edges of the cube) corresponding to the second case in Eq. (41) with piecewise-monotone structure (as shown in Sect. 3.3). Other portions of the domain (in the neighbourhood of $p_1=2.5,\ p_2=3$), however, correspond to the first case in Eq. (41), when both pools are active.

### Remark 5.5

*(Sparsity results extend to a multi-layered network)* Theorem 5.2 extends the sparsity results from the input layer to the pool layer. These sparsity results would also hold for networks with more layers.

### Remark 5.6

*(From analytical solutions/sparse piecewise structure to non-sparse LP/NP-hardness)*

Section 5 finds analytically the optimal solution for a $\mathrm{I}{+}\mathrm{H}{-}\mathrm{L}{-}1$ pooling topology with Assumption 2.2. Since the $\mathrm{I}{+}\mathrm{H}{-}\mathrm{L}{-}1$ subclass is an LP extension of the $\mathrm{I}{+}\mathrm{H}{-}1{-}1$ instance, relaxing any constraint assumption, as described in Remark 3.3.10, leads to intractable analytical solutions and vanishing sparse structure. As explained in Remark 4.6, expanding to full topology $\mathrm{I}{+}\mathrm{H}{-}\mathrm{L}{-}\mathrm{J}$ results in NP-hardness.

## Concluding remarks

This paper builds a framework analyzing standard pooling problem subclasses by parametrizing the objective function with respect to pool concentrations. The bottom-up analysis develops strongly-polynomial time algorithms for multiple pooling network topological subclasses, all in the presence of a single quality, an unbounded number of feeds to pools and also outputs (direct bypass flows) and certain flow assumptions. Patterns and hierarchies of dominating topologies are used to find active network structure. The sparsity identified in the active network structure at optimality is then linked to a pool parametric piecewise structure of the objective function. The result reveals analytically Professor Floudas’ intuition of piecewise structure in pooling problem instances.

The parametric objective function is then shown to be piecewise-monotone for instances with one output, allowing exact global solutions in strongly-polynomial time as alternatives to black-box linear programming. The insights are further used for non-linear instances with multiple outputs and one pool to overcome piecewise non-monotonicity via stationary points found in strongly-polynomial time. This result introduces a new reference point on the *P* / *NP* boundary for standard pooling subclasses, as any relaxation of assumptions or full topology (multiple pools and outputs) are shown to reach NP-hardness. The multiple outputs subclass and its assumptions includes the Haverly [33] pooling problems, showing for the first time they have exact, analytical solutions.

The position on the *P* / *NP* boundary of the multiple outputs and one pool subclass is thus an ideal starting point for approximating algorithms that cross into NP-hardness. Moreover, this paper developed intuition around sparse solutions and the conditions under which sparsity vanishes. This encourages future research in building disjunctive cuts based on the structures identified to partition feasible space in the non-sparse NP-hard subclasses, an approach taken by the state-of-the-art heuristic developed in [21].

## Omitted proofs

### Proof of Proposition 3.2.4

Lemma 3.2.3 implies w.l.o.g. that $C_i>P^U$ (the other case $C_i<P^L$ is analogous). Then $C_j<P^U$, otherwise $(C_iz_i+C_jz_j)/(z_i+z_j)>P^U$ implying $\{i,j\}$ infeasible. Two cases now arise:

1. $C_j>P^L$: Then $\gamma _i<\gamma _j$, otherwise $j\succeq \{i, j\}$. Therefore increasing the cheaper flow $z_i$ maximizes *f*, but since $C_i>C_j$, doing so also increases concentration $(C_iz_i+C_jz_j)/(z_i+z_j)$, which should not exceed $P^U$ for feasibility. Consequently, $(C_iz_i+C_jz_j)/(z_i+z_j)=P^U$.
2. $C_j<P^L$: If $\gamma _i<\gamma _j$ then, as in the previous case, $(C_iz_i+C_jz_j)/(z_i+z_j)=P^U$. Otherwise, if $\gamma _i>\gamma _j$, decreasing output concentration till bound $P^L$ and therefore increasing weight of lower cost $\gamma _j$ maximizes *f*, so $(C_iz_i+C_jz_j)/(z_i+z_j)=P^L$.

Analogously when $C_i<P^L$, if $P^L<C_j<P^U$ then $(C_iz_i+C_jz_j)/(z_i+z_j)=P^L$, or else if $C_j>P^U$ then $(C_iz_i+C_jz_j)/(z_i+z_j)=P^L$ or $P^U$ (depending on whether $\gamma _i<\gamma _j$ or $\gamma _i>\gamma _j$, respectively). Aggregating all the cases analyzed, $(C_iz_i+C_jz_j)/(z_i+z_j)=P^L$ if $(C_i-C_j)(\gamma _i-\gamma _j)>0$ and $(C_iz_i+C_jz_j)/(z_i+z_j)=P^U$ if $(C_i-C_j)(\gamma _i-\gamma _j)<0$, corresponding to *P*(*i*, *j*) defined in Eq. (12). $\square$

### Proof of Lemma 3.3.3

For Problem P–3.4, $f_{ij}=dD-x_i\gamma _i-x_j\gamma _j$ and $f_{\{ij,q\}}=dD-x'_i\gamma _i-x'_j\gamma _j- z_q\gamma _q$. Therefore,

$$\begin{aligned} ij\preceq _p\{ij,q\} \ \Leftrightarrow \ x_i\gamma _i+x_j\gamma _j\ge x'_i\gamma _i+x'_j\gamma _j+z_q\gamma _q, \end{aligned}$$

which, by replacing all flows with their solutions from Eq. (7) and Eq. (16), can be rewritten as:

$$\begin{aligned} \gamma _{ij}(p)\ge \gamma _{\{ij,q\}}(p) \ \Leftrightarrow \ \frac{D(p-P(ij,q))\big (\gamma _i(p-C_j)+\gamma _j(C_i-p)+\gamma _q(C_j-C_i)\big )}{(C_i-C_j)(p-C_q)}\ge 0.\ \end{aligned}$$

Since constant demand $D>0$ and output concentration *P*(*ij*, *q*) is a linear combination of $p,C_q$, then $D(p-P(ij,q))\big /(p-C_q)\ge 0$ and the dominance condition becomes:

$$\begin{aligned} \frac{\gamma _i(p-C_j)+\gamma _j(C_i-p)+\gamma _q(C_j-C_i)}{C_i-C_j}\ge 0 \ \Leftrightarrow \ \gamma _{ij} = \frac{\gamma _i(p-C_j)+\gamma _j(C_i-p)}{C_i-C_j}\ge \gamma _q . \end{aligned}$$

with the breakpoint achieved by solving for *p* at equality. $\square$

### Proof of Proposition 3.3.5.ii

For any breakpoint *p* between $\{i,j,q\}$ and $\{i,j,r\}$, the following must hold,

$$\begin{aligned} \{i,j,q\}\asymp _{p}\{i,j,r\}\ \overset{{\text {Eq. (3)}}}{\Leftrightarrow }\ \gamma _ix_i+\gamma _jx_j+\gamma _qz_q=\gamma _ix'_i+\gamma _jx'_j+\gamma _rz_r, \end{aligned}$$

which after replacing the flow variables on both sides from Eq. (16), becomes,

$$\begin{aligned}&\frac{\gamma _i(p-C_j)(P(ij,q)-C_q))}{(C_i-C_j)(p-C_q)}+\frac{\gamma _j(p-C_i)(P(ij,q)-C_q))}{(C_j-C_i)(p-C_q)}+\frac{\gamma _q(p-P(ij,q))}{p-C_q} \\&\quad =\frac{\gamma _i(p-C_j)(P(ij,r)-C_r))}{(C_i-C_j)(p-C_r)}+\frac{\gamma _j(p-C_i)(P(ij,r)-C_r))}{(C_j-C_i)(p-C_r)}+\frac{\gamma _r(p-P(ij,r))}{p-C_r}. \end{aligned}$$

Multiplying out the common denominator of all terms and then grouping the first two terms from each side and factoring out *p* results in:

$$\begin{aligned} \begin{aligned}&p^2(\gamma _i-\gamma _j)\big ((P(ij,q)-C_q)-(P(ij,r)-C_r)\big )\\&\quad + p\bigg (\big (P(ij,r)C_q - P(ij,q)C_r\big )(\gamma _i-\gamma _j)- (\gamma _iC_j-\gamma _jC_i) \big ((P(ij,q)-C_q)-(P(ij,r)-C_r)\big )\bigg )\\&\quad -(\gamma _iC_j-\gamma _jC_i)(P(ij,r)C_q - P(ij,q)C_r)\\&\quad + p^2(C_i-C_j)(\gamma _q-\gamma _r)+ p(C_i-C_j)\big (\gamma _rC_q+\gamma _rP(ij,r)-\gamma _qC_r-\gamma _qP(ij,q)\big )\\&\quad +(C_i-C_j)\big (\gamma _qC_rP(ij,q)-\gamma _r C_q P(ij,r)\big ) = 0. \end{aligned} \end{aligned}$$

Assuming *P*(*ij*, *q*) and *P*(*ij*, *r*) are independent of *p* results in a quadratic equation in terms of *p*,

$$\begin{aligned} {\varGamma }\big (p, P(ij,q), P(ij,r)\big ) = p^2(a_1b_1+a_3c_1)+p(a_1b_2-a_2b_1+a_3c_2)+(a_3c_3-a_2b_2)=0, \end{aligned}$$

with the following notation:

$$\begin{aligned} \begin{aligned} a_1&=\gamma _i-\gamma _j,&b_1=(P(ij,q)-C_q)&c_1=\gamma _q-\gamma _r, \\ a_2&=\gamma _iC_j-\gamma _jC_i,&\quad -(P(ij,r)-C_r),&c_2=\gamma _r(C_q+P(ij,r))-\gamma _q(C_r+P(ij,q)), \\ a_3&=C_i-C_j,&b_2= P(ij,r)C_q - P(ij,q)C_r,&c_3=\gamma _qC_rP(ij,q)-\gamma _r C_q P(ij,r). \end{aligned} \end{aligned}$$

Assuming $\Delta ^2>0$, ${\varGamma }$ has up to two roots as possible breakpoints,

$$\begin{aligned} p_{1,2}= \frac{-(a_1b_2-a_2b_1+a_3c_2)\pm \sqrt{(a_1b_2-a_2b_1+a_3c_2)^2-4(a_1b_1-a_3c_1)b_2(a_3-a_2)}}{2(a_1b_1-a_3c_1)}. \end{aligned}$$

$$\square $$

### Proof of Proposition 3.3.7

The first constraint of Problem P–3.4 implies $z_q=D-x_i-x_j$, which in turn implies,

$$\begin{aligned} \begin{aligned} f_A(p)=&d\cdot D-\gamma _ix_i-\gamma _jx_j-\gamma _qx_q=(d-\gamma _q)D- (\gamma _i-\gamma _q)x_i-(\gamma _j-\gamma _q)x_j,\\ {\frac{\partial f_A}{\partial p}} =&{\frac{\partial }{\partial p}}\left( (\gamma _i-\gamma _q)x_i+(\gamma _j-\gamma _q)x_j \right) \\ \overset{{\text {Eq. (16)}}}{=}&\frac{D\big (P(ij,q)-C_q\big )}{C_i-C_j}\cdot {\frac{\partial }{\partial p}}\bigg ( \frac{(\gamma _i-\gamma _q)(p-C_j)}{p-C_q}- \frac{(\gamma _j-\gamma _q)(p-C_i)}{p-C_q} \bigg )\\ =&\frac{D\big (P(ij,q)-C_q\big )}{C_i-C_j}\cdot \bigg ({\frac{\partial }{\partial p}}\frac{p(\gamma _i-\gamma _j)}{p-C_q} + {\frac{\partial }{\partial p}}\frac{C_i(\gamma _j-\gamma _q)- C_j(\gamma _i-\gamma _q)}{p-C_q} \bigg )\\ =&\frac{D\big (P(ij,q)-C_q\big )}{C_i-C_j}\cdot \frac{C_j(\gamma _i-\gamma _q)+ C_i(\gamma _q-\gamma _j)+C_q(\gamma _j-\gamma _i)}{(p-C_q)^2}\\ =&-\frac{D\big (P(ij,q)-C_q\big )}{(C_i-C_j)(p-C_q)^2}\cdot \bigg (C_q(\gamma _j-\gamma _i) + C_j(\gamma _i-\gamma _q)+ C_i(\gamma _q-\gamma _j)\bigg ).\\ \end{aligned} \end{aligned}$$

By deriving to further orders and due to ${\frac{\partial ^{2} f_A}{\partial p^{2}}} = \frac{-2}{(p-C_q)}{\frac{\partial f_A}{\partial p}}$, the desired assertions are obtained. $\square$
